# Antibiotic resistance in the Middle East and Southern Asia: a systematic review and meta-analysis

**DOI:** 10.1093/jacamr/dlaf010

**Published:** 2025-02-19

**Authors:** Rachel Mathu, Elizabeth Diago-Navarro, Emily Lynch, Marie-Amélie Degail, Janet Ousley, Rupa Kanapathipillai, Justine Michel, Marc Gastellu-Etchegorry, Nada Malou

**Affiliations:** Médecins Sans Frontières, New York, USA; Médecins Sans Frontières, New York, USA; Barcelona Institute for Global Health, PR3 Hub, Barcelona, Barcelona, Spain; Department of Intervention Epidemiology and Training, Epicentre, Paris, France; Department of Intervention Epidemiology and Training, Epicentre, Paris, France; Médecins Sans Frontières, Paris, France; Médecins Sans Frontières, Paris, France; Médecins Sans Frontières, Paris, France; Department of Intervention Epidemiology and Training, Epicentre, Paris, France; Médecins Sans Frontières, Paris, France

## Abstract

**Introduction:**

Despite global surveillance efforts, antibiotic resistance (ABR) is difficult to address in low- and middle-income countries (LMICs). In the absence of country-wide ABR surveillance data, peer-reviewed literature is the next most significant source of publicly available ABR data. Médecins Sans Frontières conducted this review in hopes of using the pooled findings to inform treatment choices in the studied countries where sufficient local ABR data are unavailable.

**Methods:**

A systematic literature review reporting ABR rates for six infection sites in nine countries in the Middle East and Southern Asia was conducted. PubMed was used to identify literature published between January 2012 and August 2022. A meta-analysis of the included studies (*n* = 694) was conducted, of which 224 are reviewed in this paper. The JBI critical appraisal tool was used to evaluate risk of bias for included studies.

**Results:**

This paper focuses on sepsis, burns and wound infections, specifically, with the largest number of papers describing data from Iran, Türkiye and Pakistan. High (>30%) resistance to recommended first-line antibiotics was found. Gram-negative resistance to ceftriaxone, aminoglycosides and carbapenems was high in burn-related infections; colistin resistance among *Klebsiella pneumoniae* isolates in Pakistan was alarmingly high (81%).

**Conclusions:**

High-quality data on ABR in LMIC settings remain difficult to obtain. While peer-reviewed literature is a source of publicly available ABR data, it is of inconsistent quality; the field also lacks agreed reporting standards, limiting the capacity to pool findings. Nonetheless, high resistance to first-line antibiotics underscores the need for improved localized surveillance and stewardship.

## Introduction

Increasing levels of antibiotic resistance (ABR) jeopardize critical preventive and therapeutic tools for infectious diseases.^1^ In some high-burden regions—including countries in the Middle East and Southern Asia—this challenge is further complicated by weak surveillance systems, a lack of sufficient or usable ABR data and health systems affected by conflict or systemic ABR risk factors.^2^ Although some ABR surveillance occurs in these regions, high-quality, publicly available data at regional, national and hospital levels remain scarce. As of June 2022, 21 of 22 countries in the Arab League were part of the WHO's Global ABR Surveillance System (GLASS), though country-level reporting is widely variable and can be inconsistent and lack uniformity (reporting mechanisms can also be incompletely implemented in this region).^3^ This potentially compromises the utility of such a system, both for policymakers and for the revision of empiric treatment guidelines.

As ABR increases and diversifies, Médecins Sans Frontières (MSF) is challenged to implement effective treatment for critical patients, from war-wounded patients in Iraq, Syria, Yemen and Palestine to neonates in Afghanistan and Pakistan. In the context of clinical deterioration with sepsis, clinicians must empirically escalate antibiotic treatments when needed.^2^ In these circumstances, patient management relies primarily on clinical guidelines and empirical treatment, which may not be adapted or updated due to insufficient ABR data, with little to no ability of ABR stewardship to respond to emergent resistance. Devi *et al*.^4^ call this lack of sufficient ABR information in conflict-affected populations the ‘perfect storm’ to exacerbate antimicrobial resistance.

Local surveillance using hospital-level data via hospital antibiograms is critical to guide locally appropriate patient management.^1^ Hospital antibiograms are a periodic summary of the antimicrobial susceptibilities to isolated pathogens in a specific population, in a specific area. They are created using clinical microbiology laboratory data and are used by clinicians to select and update empirical therapy and surgical prophylaxis, to monitor bacterial resistance trends over time and to perform surveillance of drug resistant organisms and identify possible interventions and stewardship programmes.^5^ Access to microbiological laboratory services and sufficient clinical microbiological expertise is a key to reducing ABR and to informing national policy and empiric guidelines at the hospital level.^6,7^

This systematic review and meta-analysis was originally conducted by MSF for the countries of interest to help clinical teams select the best empiric antibiotics available based on their region, patient population and the highest priority infectious syndromes. This research expands a 2018 systematic review of ABR evidence in the Middle East previously published^8^ by providing a meta-analysis of the sometimes widely varying ABR figures for these countries, including similarly conflict-affected countries in Southern Asia and stratifying information by pathogen, type of resistance and infection site.

## Methods

This systematic review and meta-analysis was performed in accordance with 2020 Preferred Reporting Items for Systemic Reviews and Meta-Analyses (PRISMA) guidelines.^9^ The complete PRISMA checklist is included as a supplementary table (Table S1, available as Supplementary data at *JAC-AMR* Online). Our geographic region of interest focused on nine countries (Iraq, Syria, Lebanon, Yemen, Palestine, Türkiye, Iran, Afghanistan and Pakistan), of which five (Iraq, Syria, Lebanon, Palestine and Yemen) were also featured in the previous systematic review in 2018.^8^ As this current review was motivated primarily by the urgency of operational research questions, we focused on low- and middle-income countries in the Middle East and Southern Asia with active MSF medical facilities in either conflict-affected regions or with facilities serving conflict-affected populations.

The researchers identified relevant literature published between 1 January 2012 and 31 August 2022 via PubMed and through backward citation searches of identified literature. A meta-analysis of the results per country, per pathogen and per infection site was conducted.

Terms used in the search were as follows: (UTI resistance OR UTI sensitivity OR Urinary Tract Infection resistance OR Urinary Tract sensitivity OR UTI antibiotics or urinary tract infection antibiotics OR blood culture antibiotics OR blood culture resistance OR blood culture sensitivity OR sepsis antibiotics OR sepsis resistance OR sepsis sensitivity OR bacteraemia antibiotics OR bacteraemia resistance OR bacteraemia sensitivity OR Osteomyelitis antibiotics OR osteomyelitis sensitivity OR bone antibiotic resistance OR bone infection antibiotics OR bone infection resistance OR bone infection sensitivity OR tissue antibiotic resistance OR tissue infection antibiotics OR tissue infection resistance OR tissue infection sensitivity OR wound infection antibiotic resistance OR wound infection resistance OR wound infection sensitivity) AND (specific country).

### Inclusion and exclusion criteria

Publications were reviewed by one investigator and were discussed with at least one additional investigator to determine their eligibility. Data were collected by one investigator. Included papers were in English (or had a comprehensive abstract in English), identified bacterial causes of infection and ABR rates and described rates in any of the following sites of infection: urinary tract infections, bloodstream infection (BSI)/sepsis, bone infections, wound infections, burn infections and infections of mixed origins. For this paper, the authors have chosen to focus on the results for BSI/sepsis, burns, and wound-related infections. The data on these infection sites are both high priority to MSF clinical questions and operations and, for the countries of interest, contained relatively more data than other infection sites.

Information on other infection sites is available upon reasonable request from the corresponding author.

**Figure 1.**
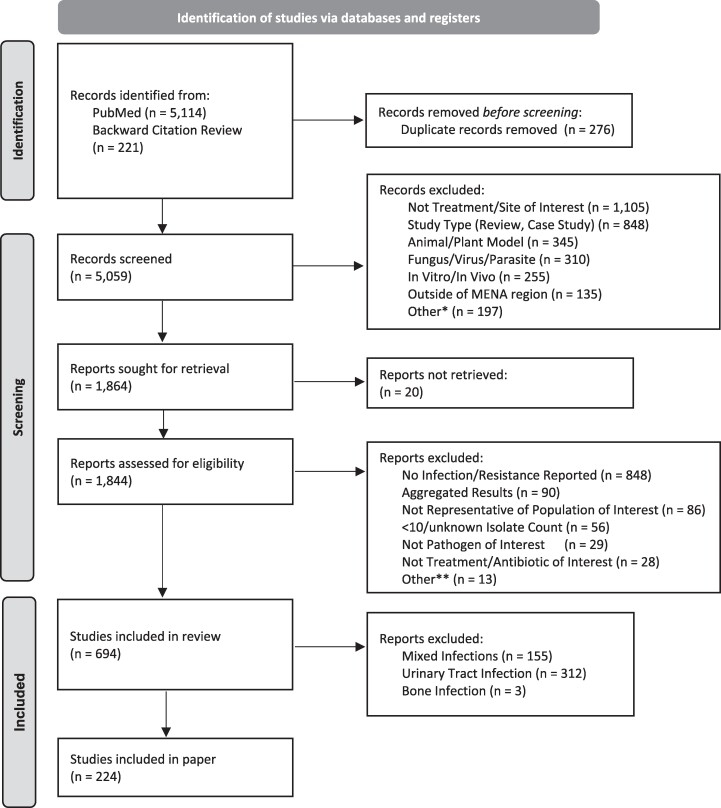
PRISMA flow of included/excluded articles. *Not in english, colonization, retracted study. **Questionable results, not disease/site of interest, multiple isolates per person.

To minimize bias, studies with <10 patients were excluded, as were those with <10 isolates for all reported species, a slightly less rigorous standard than the <30 isolates required per the standards of the Clinical and Laboratory Standard Institute, but one which allows for potentially clinically relevant results to still be described.^5^ Studies reporting aggregate data (such as resistance rates for Gram-negative organisms) or more than one country were excluded. Multi-country studies were included when data were disaggregated by country. Research combining environmental or animal samples, in addition to human samples, was excluded unless resistance was disaggregated by type of sample. Studies reporting a mixture of specimen sources without disaggregation of the data by specimen type (e.g. urine plus blood) were included in a ‘mixed infections’ category, which is not reviewed in this paper. Wound infections combine wound and skin and soft tissue infections. For wound and burn sites, we included samples from sterile and intra-operative sites and swabs. We excluded data derived from multiple specimens from one patient. We collected standardized information on the number of samples, study methodologies, limitations, populations, bacteria isolated and ABR resistance [focusing on extended-spectrum beta-lactamases (ESBL), methicillin-resistant *Staphylococcus aureus* (MRSA) and carbapenemase-producing bacteria and/or resistance to the following: ceftriaxone, amikacin, ciprofloxacin, gentamicin, imipenem/meropenem, vancomycin and colistin/polymyxin B]. We included both retrospective and prospective studies from hospitals, clinics or surveillance studies. When available, information on cohort age was also captured and categorized into adult (≥18), paediatric (<18) or all ages. The study inclusion process is outlined in Figure 1.

### Quality assessment

The study evaluated the methodological quality of eligible papers using the JBI Checklist for Prevalence Studies, which scores quality in nine parts to determine if biases have been addressed in its design, conduct and analysis.^10^ Question 9 about the response rate was found not applicable for our studies, thus only eight questions were addressed. Papers were reviewed by one investigator and confirmed by another. The other questions reviewed the sufficiency of the (i) sampling frame, (ii) recruitment of participants, (iii) sample size, (iv) description of population and setting, (v) data analysis for coverage, (vi) differential diagnosis/methods, (vii) standardized assessment and (viii) statistical analysis. Table S2 provides more detail on how the authors applied each question to the content of this study.

### Data analysis

This paper reports only ABR rate data derived from papers reporting ≥10 isolates per species per paper with a combined total of a minimum of at least 30 isolates per species. Within each infection diagnosis, the point prevalence and 95% confidence interval (CI) were calculated for each pathogen–antimicrobial pair. Additionally, data were summarized by country, type of infection and organism (with number of isolates included) for each of the antibiotics of interest and presented in tables. For infection sites and countries with substantial isolate counts (>30 aggregated isolates), results were stratified by paediatric and non-paediatric (e.g. adult, all ages) populations. High resistance was defined as resistance >30%.^11^ Data analysis was performed using transformation of the proportions via the Freeman–Tukey double arcsine method and then meta-analysed with an inverse-variance–weighted random-effects analysis. STATA version 18.0 was used for the analysis.^12^

## Results

### Overview of study characteristics

A total of 5144 articles were found using 29 search terms for the 9 countries and for high priority infectious clinical syndromes most relevant for MSF operations (see ‘Inclusion and exclusion criteria’) with an additional 221 articles identified through background citation review. Of the identified studies, 1844 were assessed for eligibility and ultimately 694 were included (of which 224 are described in this paper) (Figure 1).

Among the 694 papers, the largest number of studies/papers focused on data from 4 countries: Iran (*n* = 305, 43.9%), Pakistan (*n* = 145, 20.9%), Türkiye (*n* = 114, 16.4%) and Iraq (*n* = 69, 9.9%).

This manuscript focuses on sepsis, burns and wound-related infections and discusses 224/694 studies, including 122 (54.5%) studies on sepsis, 62 (27.7%) studies on burns and 48 (21.4%) studies on wound-related infections. Of the 224 studies, 8 reported on more than one infection type and were counted only once in the study total. Descriptive characteristics of the included studies are presented in Table 1.

**Table 1.** General characteristics of studies included in the meta-analysis

	Site of infection^13–236^			
	All sites (*N* = 224)	Bloodstream (sepsis) (*N* = 122)	Burn related (*N* = 62)	Wound related (*N* = 48)
Countries with studies, *n* (%)				
Afghanistan	1 (0.5)	1 (0.8)	0 (0.0)	0 (0.0)
Iran	75 (33.5)	28 (23.0)	37 (59.7)	13 (27.1)
Iraq	17 (7.6)^a^	5 (4.1)^a^	8 (12.9)	4 (8.3)
Lebanon	5 (2.2)	2 (1.6)	1 (1.6)	2 (4.2)
Pakistan	64 (28.6)^a^	42 (34.4)^a^	9 (14.5)	16 (33.3)
Palestine	5 (2.2)	1 (0.8)	2 (3.2)	2 (4.2)
Syria	2 (0.9)	0 (0.0)	1 (1.6)	1 (2.1)
Türkiye	53 (23.7)	43 (35.2)	3 (4.8)	7 (14.6)
Yemen	3 (1.3)	1 (0.8)	1 (1.6)	3 (6.3)
Age of population studied, *n* (%)				
Adult	32 (14.3)	22 (18.0)	4 (6.5)	6 (12.5)
Paediatric	49 (21.9)	45 (36.9)	1 (1.6)	4 (8.3)
All ages	65 (29.0)	26 (21.3)	26 (41.9)	17 (35.4)
Undefined	78 (34.8)	29 (23.8)	31 (50.0)	21 (43.8)
Patient status, *n* (%)				
Inpatient	149 (66.5)	87 (71.3)	43 (69.4)	22 (45.8)
Outpatient	7 (3.1)	1 (0.8)	0 (0.0)	6 (12.5)
Inpatient/outpatient	16 (7.1)	5 (4.1)	2 (3.2)	12 (25.0)
Undefined	52 (23.2)	29 (23.8)	17 (27.4)	8 (16.7)
Type of study, *n* (%)				
Cross-sectional	214 (95.5)	116 (95.1)	62 (100)	44 (91.7)
Cohort	6 (2.7)	3 (2.5)	0 (0.0)	3 (6.3)
Case–control	3 (1.3)	2 (1.6)	0 (0.0)	1 (2.1)
Surveillance	1 (0.5)	1 (0.8)	0 (0.0)	0 (0.0)
Type of microbiology lab, *n* (%)				
Clinical/hospital	184 (82.1)	106 (86.9)	60 (96.8)	23 (47.9)
Academic/research	39 (17.4)	15 (12.3)	2 (3.2)	25 (52.1)
Private	1 (0.5)	1 (0.8)	0 (0.0)	0 (0.0)
Susceptibility test method, *n* (%)				
Disc diffusion	146 (65.2)	71 (58.2)	52 (83.8)	30 (62.5)
Mixed methods	30 (13.4)	16 (13.1)	5 (8.1)	10 (20.8)
Other/undefined	48 (21.4)	35 (28.7)	5 (8.1)	8 (16.7)
Clinical guidelines followed, *n* (%)				
CLSI	165 (73.7)	85 (69.7)	55 (88.7)	30 (62.5)
Other (EUCAST, NCCLS, etc.)	11 (4.9)	7 (5.7)	1 (1.6)	3 (6.3)
Undefined	48 (21.4)	30 (24.6)	6 (9.7)	15 (31.3)

CLSI, Clinical and Laboratory Standards Institute; EUCAST, European Committee on Antimicrobial Susceptibility Testing; NCCLS, National Committee for Clinical Laboratory Standards.

^a^A study reported resistance rates stratified by country (Iraq and Pakistan). Therefore, percentages associated with country counts add to more than 100%.

**Figure 2.**
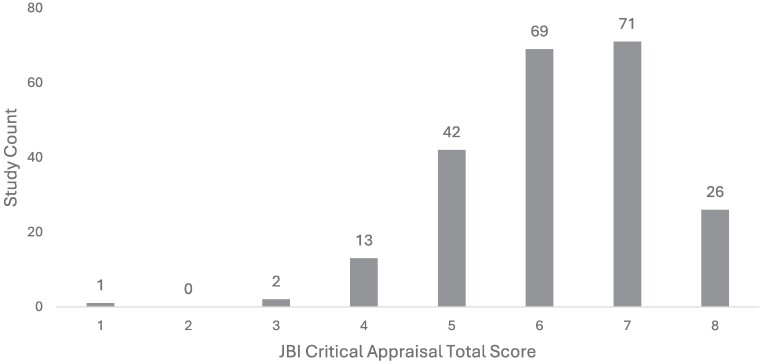
Distribution of study quality by JBI Critical Appraisal score (higher = better; *n* = 224).

Overall, methodological quality of the papers included in the study was reasonable but not excellent. The JBI quality assessment of the 224 papers showed a median score of 6/8 (75%). Papers reporting data from Lebanon, Pakistan and Palestine had the lowest overall methodological quality, whereas those reporting data from Yemen, Syria and Iran had the highest quality, followed closely by data from Iraq and Turkey. Only 26 studies met all the criteria (8/8), and only one paper met a single criterion (1/8, 13%). Two studies met 3/8 (38%) criteria, and 13 papers met 4/8 (50%) (Figure 2). Many studies (*n* = 90/224, 40%) did not meet the standard set by Criterion 3 for sufficient sample size (i.e. a minimum of 10 isolates reported for any organism), nor did many sufficiently describe the population or setting as defined in Criterion 4 (*n* = 100/224, 45%).

### Resistance rates by infection site and bacterial pathogen

#### Sepsis

Studies of bloodstream infections (sepsis) (*n* = 122) came predominantly from Türkiye (*n* = 43), Pakistan (*n* = 42) and Iran (*n* = 28), with 36.9% of these focused on paediatric populations and 71.3% reporting on hospitalized patients. A total of 86.9% of the studies used a clinical/hospital laboratory to analyse resistance. Studies were largely cross-sectional clinical research [116 (95.1%)] (Table 1).

**Figure 3.**
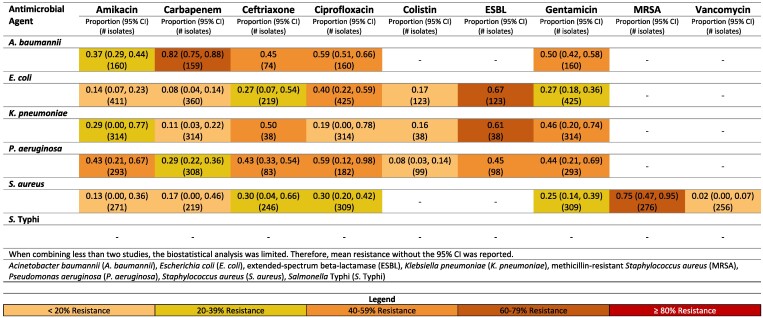
Resistance patterns of *A. baumannii*, *E. coli*, *K. pneumoniae*, *P. aeruginosa, S. aureus* and *Sa.* Typhi isolates found in blood stream infections (sepsis) to the antimicrobial agents of interest in Iran.^13–29^

**Figure 4.**
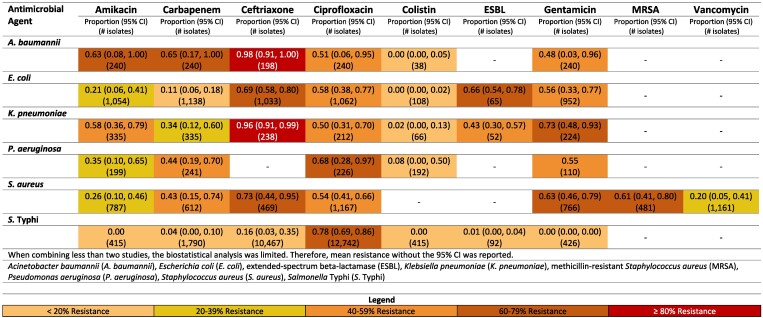
Resistance patterns of *A. baumannii*, *E. coli*, *K. pneumoniae*, *P. aeruginosa, S. aureus* and *Sa.* Typhi isolates found in blood stream infections (sepsis) to the antimicrobial agents of interest in Pakistan.^30–59,167,180,182,192,207,229,233,235^

**Figure 5.**
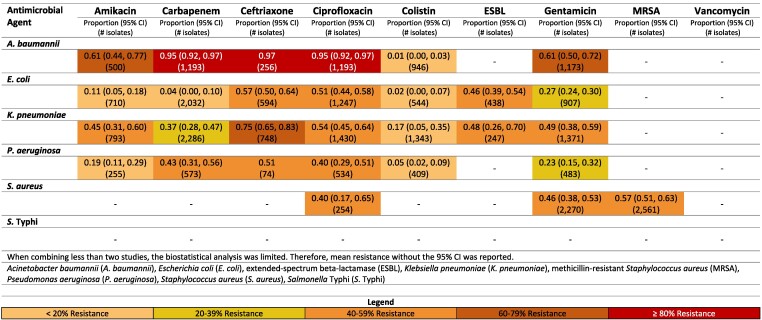
Resistance patterns of *A. baumannii*, *E. coli*, *K. pneumoniae*, *P. aeruginosa, S. aureus* and *Sa. T*yphi isolates found in blood stream infections (sepsis) to the antimicrobial agents of interest in Türkiye.^60–80,145,148,150,159,172,174^

Among the Gram-negative pathogens, the most common pathogens were *Salmonella* Typhi (29.7%, *n* = 12 825), *Escherichia coli* (9.1%, *n* = 3917), *Klebsiella pneumoniae* (7.2%, *n* = 3103), *Acinetobacter baumannii* (4.2%, *n* = 1809), and *Pseudomonas aeruginosa* (2.9%, *n* = 1254) (Table 2). For Gram-positive pathogens, *Staphylococcus aureus* was the most frequently reported (11.3%, *n* = 4883) (Table 2). Figures reporting resistance rates for Türkiye, Pakistan and Iran are available in-text (Figures 3–5). All additional figures are available in supplementary data (Figures S1–S9).

**Table 2.** Frequent pathogens reported in included studies stratified by site of infection^13–236^

	Bloodstream (sepsis) (*N *= 43 176 isolates)	Burn related (*N* = 8738 isolates)	Wound related (*N*= 5018 isolates)
Pathogens			
* A. baumannii*	1809 (4.2%)	1399 (16.0%)	91 (1.8%)
* E. coli*	3917 (9.1%)	287 (3.3%)	445 (8.9%)
* K. pneumoniae*	3103 (7.2%)	306 (3.5%)	86 (1.7%)
* P. aeruginosa*	1254 (2.9%)	3229 (37.0%)	378 (7.5%)
* S. aureus*	4883 (11.3%)	1405 (16.1%)	2981 (59.4%)
* Sa.* Typhi	12 825 (29.7%)	—	—
* *Other pathogens^a^	15 385 (35.6%)	2112 (24.2%)	1037 (20.7%)

^a^Other pathogens include Acinetobacter species, Citrobacter species, Enterobacter species, Klebsiella species, Proteus species, Streptococcus species, etc. The most frequent or clinically important species were reported above.

##### Iran

Carbapenem resistance of *A. baumannii* was 82% (95% CI: 75%–88%) (Figure 3).^13,14^ Resistance to carbapenem for *E. coli* was noticeably lower [8% (95% CI: 4–14%)] in Iran.^13,15–18^

A high percentage of *S. aureus* isolates in Iran were methicillin resistant [75% (95% CI: 47%–95%)].^17,19–22^ This was largely driven by the non-paediatric studies reporting an MRSA rate of 94% (95% CI: 90%–97%)^19,22^ compared with 69% (95% CI: 57%–80%) in the paediatric population.^17,20,21^ Vancomycin resistance was reported with low resistance rates of 2% (95% CI: 0%–7%) in Iran.^15,19,21,23^

Rates of resistance of *E. coli* to ceftriaxone were higher in non-paediatric [46% (95% CI: 38–54)]^18,24^ compared with paediatric [25% (95% CI: 1–63)].^15,17,25^ Interestingly, carbapenem resistance was comparable for Gram negatives comparing non-paediatric to paediatric studies in Iran.^13–18,26–29^

##### Pakistan

Ceftriaxone resistance in Pakistan to *K. pneumoniae* was notably high at 96% (95% CI: 91–99%).^30,31,180,182,192,233^ Pakistan also reported low rates of carbapenem resistance to *E. coli* [11% (95% CI: 6–18%)].^32–40,233^

Pakistan had substantial literature reporting on resistance to *Sa.* Typhi (Figure 4). Resistance rates <16% were seen for all reported microbial agents other than ciprofloxacin, which had a relatively high resistance rate of 78% (95% CI: 69–86%).^41–55,207,233^ Ciprofloxacin-resistant *Sa.* Typhi was higher in the non-paediatric population [83% (95% CI: 74–90%)]^41–44,46–49,52,53,55,207^ (Figure S3), when compared with the paediatric population [59% (95% CI: 41%–76%)] (Figure S4).^45,50,51,54,233^

Pakistan reported moderately high rates of MRSA 61% (95% CI: 41–80%).^38,40,56,57,182,233^ Vancomycin resistance in *S. aureus* was reported 20% (95% CI: 5–41%) in Pakistan.^30,32,35,36,38,40,50,56–58,182,233^

MRSA rates were higher in paediatric studies from Pakistan [62% (95% CI: 41–82%)]^40,57,182,233^ (Figure S4) compared with non-paediatric studies [38% (95% CI: 28–49)] (Figure S3).^38,56^  *K. pneumoniae* resistant to ceftriaxone were comparably high in paediatric (97% (95% CI: 92–100)^30,180,182,192,233^ compared with non-paediatric studies (90%).^31^  *A. baumannii* resistance to carbapenems was substantially higher in non-paediatric studies from Pakistan (90%)^59^ compared with paediatric [55% (95% CI: 2–100)].^40,180,182^

##### Türkiye

Carbapenem resistance of *A. baumannii* was 95% (95% CI: 92–97%) for Türkiye (Figure 5).^60–63,145^ Resistance to carbapenem was noticeably lower for *E. coli*, with a rate of 4% (95% CI: 0–10%) in Türkiye.^60–74^

Türkiye reported moderately high rates of MRSA [57% (95% CI: 51–63%)].^65,67,75–80^

In studies from Türkiye, rates of MRSA, *E. coli* and *K. pneumoniae* resistant to ceftriaxone were comparably high in non-paediatric and paediatric studies (Figures S5 and S6).

##### Other countries

Resistance rates were explored for other countries of interest (e.g. Afghanistan, Iraq, Lebanon, etc.) (Figures S7–S9); however, it was difficult to draw meaningful conclusions due to the limited number of isolates reported. There were a few countries reporting on pathogens with sufficient (*n* > 30) isolates that are worth noting. In Lebanon, *A. baumannii* had high rates of resistance to ciprofloxacin (96%), carbapenem (91%) and amikacin (90%).^81^ It should be noted that these rates came from one paper reporting on *n* = 90 isolates from a hospitalized, all ages population identified in a tertiary care facility in Beirut. Both Afghanistan and Lebanon had adequate isolates to report rates of MRSA, which were 65%^82^ and 45% (95% CI: 38–52%),^83^ respectively.

#### Burn infections

Studies of burn infections (*n* = 62) came predominantly from Iran (*n* = 37), Pakistan (*n* = 9), Iraq (*n* = 8) and Türkiye (*n* = 3), with 69.4% of these focused on hospitalized patients. Population age was poorly defined (50% of included studies did not define the target age group); therefore, stratified results are not available. Sampling methods were also not well described (i.e. biopsy, wound swab, etc.); therefore, studies reporting on samples of lower reliability could not be excluded. All studies were clinical cross-sectional studies. Of studies that included the type of microbiology laboratory used, the majority (96.8%) were hospital/clinical routine laboratories, with the remaining two studies being conducted at academic research labs. The studies tested resistance with the Kirby–Bauer disc diffusion test (83.8%) or other independent or mixed methods (16.2%). The majority (88.7%) of studies reported following Clinical and Laboratory Standards Institute (CLSI) guidelines. (Table 1)

**Figure 6.**
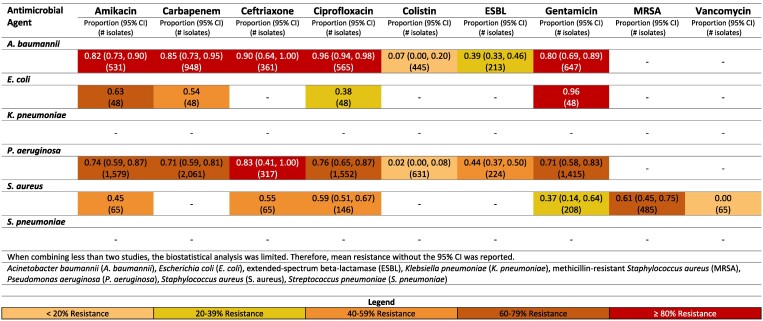
Resistance patterns of *A. baumannii*, *E. coli*, *K. pneumoniae*, *P. aeruginosa, S. aureus* and *Str. pneumoniae* isolates found in burn-related infections to the antimicrobial agents of interest in Iran.^84–97,157,169,170,175,178,188,190,197,201,202,204,208,210,218,223,225,230^

**Figure 7.**
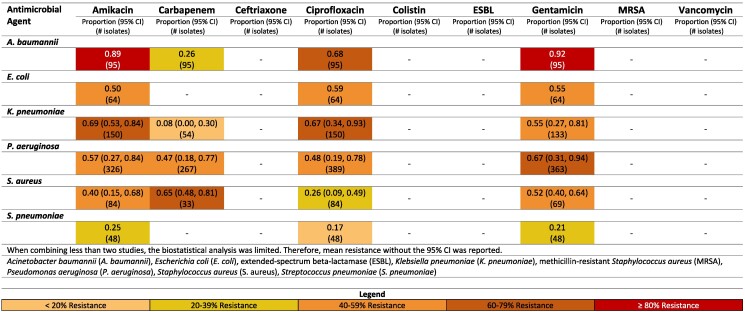
Resistance patterns of *A. baumannii*, *E. coli*, *K. pneumoniae*, *P. aeruginosa, S. aureus* and *Str. pneumoniae* isolates found in burn-related infections to the antimicrobial agents of interest in Iraq.^98,161,181,186,199,222,234^

**Figure 8.**
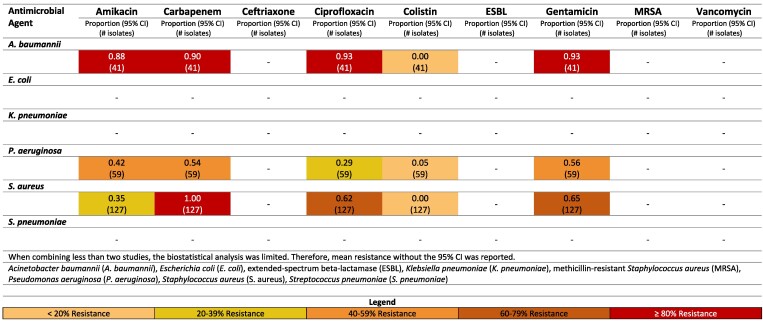
Resistance patterns of *A. baumannii*, *E. coli*, *K. pneumoniae*, *P. aeruginosa, S. aureus* and *Str. pneumoniae* isolates found in burn-related infections to the antimicrobial agents of interest in Lebanon.^99^

**Figure 9.**
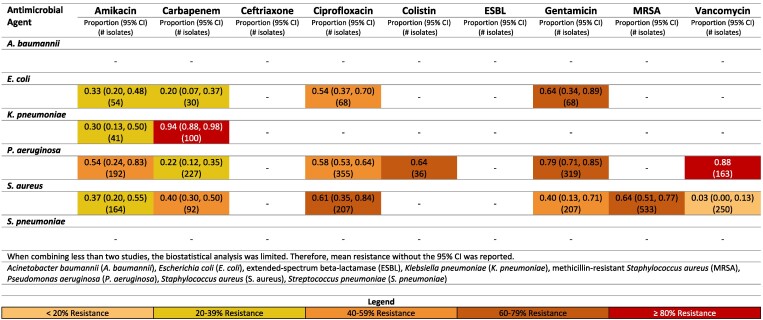
Resistance patterns of *A. baumannii*, *E. coli*, *K. pneumoniae*, *P. aeruginosa, S. aureus* and *Str. pneumoniae* isolates found in burn-related infections to the antimicrobial agents of interest in Pakistan.^100–108^

**Figure 10.**
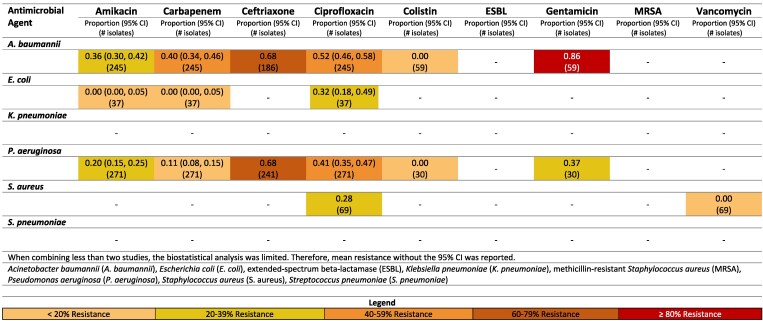
Resistance patterns of *A. baumannii*, *E. coli*, *K. pneumoniae*, *P. aeruginosa, S. aureus* and *Str. pneumoniae* isolates found in burn-related infections to the antimicrobial agents of interest in Türkiye.^109,110,176^

Among the Gram-negative pathogens the most common pathogens were *P. aeruginosa* (37.0%, *n* = 3229), *A. baumannii* (16.0%, *n* = 1399), *K. pneumoniae* (3.5%, *n* = 306) and *E. coli* (3.3%, *n* = 287). The Gram-positive pathogen most frequently reported was *S. aureus* (16.1%, *n* = 1405) (Table 2). Figures reporting resistance rates for Iran, Iraq, Lebanon, Pakistan and Türkiye are available in-text (Figures 6–10) and in supplementary data (Figure S10) for Yemen.

##### Iran

*A. baumannii* and *P. aeruginosa* registered high rates of resistance across all antimicrobial agents in Iran, except colistin, with reported resistance rates of 7% (95% CI: 0%–20%)^84–89^ and 2% (95% CI: 0%–8%),^90–96^ respectively (Figure 6).

Vancomycin resistance was 0% in Iran.^97^

##### Iraq

Iraq also reported high rates of resistance of *A. baumanii* to multiple antimicrobial agents (Figure 7). Resistance to gentamicin was reported at 92% and amikacin at 89%.^98^

##### Lebanon

High rates of resistance of *A. baumannii* to multiple antimicrobial agents was also reported (Figure 8). Gentamicin resistance of *A. baumannii* was 93%,^99^ and amikacin resistance was 88%.^99^

Moderate rates of resistance for *S. aureus* to ciprofloxacin were reported in Lebanon (62%).^99^

##### Pakistan

Pakistan had sufficient and meaningful data to report on two pathogens: *E. coli and K. pneumoniae* (Figure 9). Moderate to low resistance was seen across microbial agents by *E. coli*. The greatest resistance was to gentamicin, with 64% (95% CI: 34–64%) of *E. coli* isolates resistant.^100–103^ For *K. pneumoniae*, substantial resistance to carbapenem was identified [94% (95% CI: 88%–98%)].^104,105^

Methicillin resistance by *S. aureus* rate was reported 64% (95% CI: 51%–77%) in Pakistan,^102–108^ while vancomycin resistance was low [3% (95% CI: 0–13%)].^100–103^ Moderate rates of resistance for *S. aureus* to ciprofloxacin were reported in Pakistan [61% (95% CI: 35–84%)].^100–102,106^

##### Türkiye

Gentamicin resistance of *A. baumannii* was 86%,^109^ while it reported a lower rate for amikacin [36% (95% CI: 30–42%)] (Figure 10).^109,110^

##### Other countries

Information for Palestine and Yemen was extremely limited, limited to one study each, with the Palestinian study having an insufficient (<30) isolate count for meaningful results. The Palestinian study reported an ESBL positivity rate of 13% for 15 *P. aeruginosa* isolates. Patients in this study were hospitalized and treated at one of the two governmental hospital burn units in the Gaza Strip.^111^ In Yemen, the identified study had sufficient isolates (*n* > 30) of *P. aeruginosa* and reported concerning rates of resistance to gentamicin (87%), amikacin (83%) and ciprofloxacin (65%) (Figure S10).^112^

#### Wound-related infections

Studies reporting on wound infections (*n* = 48) focused on Pakistan (*n* = 16), Iran (*n* = 13), Türkiye (*n* = 7), Yemen (*n* = 3) and Palestine (*n* = 2), with 12.5% of studies focused on adults and 45.8% drawing data from hospitalized patients. Of the studies that included information on methodology, studies were primarily cross-sectional (91.7%) with few cohort studies available (6.3%), one case–control and no surveillance studies. Of the studies that specified the type of microbiology laboratory used, 23 (47.9%) used routine hospital/clinical laboratories and 25 (52.1%) used academic/research laboratories. Results of ABR were mostly defined by Kirby–Bauer disc diffusion [30 (62.5%)], while 10 (20.8%) studies used mixed methods. The majority of studies (62.5%) followed CLSI guidelines (Table 1).

**Figure 11.**
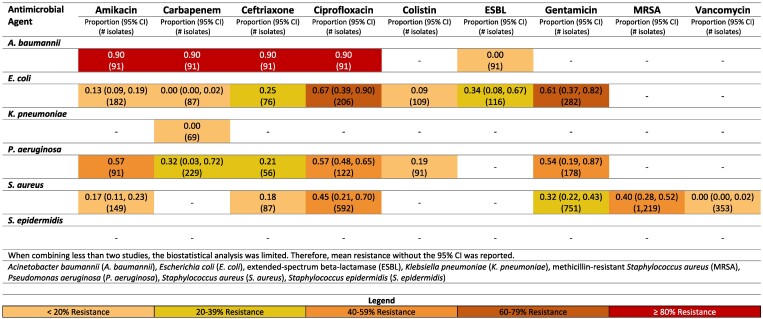
Resistance patterns of *A. baumannii*, *E. coli*, *K. pneumoniae*, *P. aeruginosa, S. aureus* and *S. epidermidis* isolates found in wound-related infections to the antimicrobial agents of interest in Pakistan.^105,107,108,113–124,215^

Among the pathogens identified, the most common were *S. aureus* (*n* = 2,981, 59.4%). Other frequent pathogens included *E. coli* (*n* = 445, 8.9%), *P. aeruginosa* (*n* = 378, 7.5%), *A. baumannii* (*n* = 91, 1.8%) and *K. pneumoniae* (*n* = 86, 1.7%) (Table 2). An in-text figure reporting resistance rates is available for Pakistan (Figure 11). All additional figures are available in supplementary data (Figures S11–S15).

Studies reporting on Gram-negative pathogens with sufficient (*n* > 30) isolates were limited to Pakistan (Figure 11) and Yemen (Figure S15).

##### Pakistan

Resistance of *A. baumannii* to antimicrobial agents in Pakistan was high, though these findings are limited to one study reporting 90% resistance to amikacin, carbapenem, and ciprofloxacin.^113^ Resistance of *P. aeruginosa* to ciprofloxacin was reported 57% (95% CI: 48–65%).^114,115^

For *E. coli*, 34% (95% CI: 8%–67%) of isolates were ESBL-producing Enterobacterales.^116,117^ Resistance to ciprofloxacin and gentamicin was moderately high [67% (95% CI: 39–90%)^115,117–119^ and 61% (95% CI: 37–82%),^115,117–120^ respectively]. Carbapenem resistance was 0% (95% CI: 0–2%),^115,117^ with amikacin [13% (95% CI: 9–19%)]^117–119^ and colistin (9%)^118^ also reporting low rates of resistance.

MRSA was reported 40% (95% CI: 28–52%).^105,107,108,117–119,121–124^  *S. aureus* resistance to gentamicin was 32% (95% CI: 22–43%) in Pakistan,^115,117,119,120,122,123^ while ciprofloxacin resistance was 45% (95% CI: 21–70%).^115,119,122,123^

##### Yemen

In Yemen, *P. aeruginosa* resistance to ciprofloxacin was reported at 69% (95% CI: 56–80),^112,125^ while amikacin resistance was notably high (83%).^112^

##### Other countries

Two Syrian studies identified, 1 with sufficient (*n* > 30) isolates and 1 with 15 isolates, reported on MRSA, at 59% (95% CI 45%–73%) (Figure S13).^126,127^

Sufficient data on *S. aureus* were available for Iran, Iraq, Türkiye, Syria and Yemen (Figures S11–S15). The rate of MRSA ranged from 65% (95% CI: 52%–76%)^20,22,128–135^ in Iran to 25% (95% CI: 11%–42%) in Türkiye.^136–140^ Vancomycin resistance was 0% in all reporting countries except Syria, which did not report on vancomycin resistance.^115,117–119,123,125,128,129,136,141–144,217^  *S. aureus* resistance to gentamicin varied quite drastically between 32% (95% CI: 22–43%) in Pakistan^115,117,119,120,122,123^ and 5% (95% CI: 0–38%) in Türkiye.^136,139,217^ A similar range for ciprofloxacin was also noted, at 45% (95% CI: 21–70%)^115,119,122,123^ and 1% (95% CI: 0–4%),^136,217^ respectively.

## Discussion

The purpose of this meta-analysis was to aid MSF clinicians in selecting the most appropriate empiric treatments for patients with severe infections in contexts lacking sufficient localized ABR data or microbiological capacity to implement targeted treatment.

The study found that data from Iran, Pakistan and Türkiye are disproportionately represented in the literature on ABR from this region. Methodological quality, according to the JBI Checklist for Prevalence Studies, was overall reasonable but not excellent, underscoring the risk of bias introduced by insufficiently rigorous methods in the original studies; this risk is elevated for papers from Lebanon, Pakistan and Palestine. For Pakistan, at least, the risk of this bias affecting interpretation of the results is offset by the large number of papers (*n* = 64), most of which (*n* = 56/64, 89%) had JBI scores of 6 or higher (out of 8), as well as the number of isolates analysed, only *A. baumannii* resistance to colistin was assessed with <50 isolates. Considering the number of isolates (methodological variation and limitations notwithstanding), sepsis is best described for Iran, Pakistan and Türkiye. Burns infections are best described for Iran, Pakistan, Iraq and Türkiye, and the data on wound infections are most abundant from Pakistan, Iran, Türkiye, Yemen and Palestine, all in descending order.

This literature review underscores what is certainly not surprising but nonetheless relevant to note for researchers and clinicians, that those countries more likely to be represented in the literature are similarly more likely to have less or no active conflict and perhaps therefore more consistent resources for antimicrobial stewardship and ABR surveillance. This finding highlights that published literature is not currently able to address the knowledge gap created by the lack of microbiological capacity and ABR surveillance in country.

Moreover, our study demonstrates the limits of making clinical use of these findings given the very poor completeness of reported data in this review around key aspects needed for clinical interpretation of the results; notably, the age of the population studied was undefined in 35% of papers (50% for those describing burns infections), inpatient versus outpatient status was undefined in 23% of papers and remarkably, the method for susceptibility testing was not defined in 21% of papers (28% of papers describing sepsis infections) and which clinical guidelines were used was undefined in 21% of papers (31% in those describing wound infections), despite 82% of studies reporting data from clinical/hospital labs. Given the financial and human resource investment required to improve country-level surveillance data globally, peer-reviewed literature remains a significant source of publicly available AMR data for LMICs in the interim. However, the lack of standardized reporting frameworks limits its clinical usability. Developing standards and guidelines for peer-reviewed reporting of AMR data, including detailed patient information and susceptibility statuses, could enhance its use for clinical care and go some way towards addressing the lack of sufficient surveillance data and widespread microbiological capacity in high priority countries.

Despite the challenges in data quality uncovered by this review, the authors note that among sepsis, burns and wound-related infections, a high rate of ABR was found across all countries in the analysis, though rates varied substantially between countries and between pathogens.

### Geographic and pathogen-specific resistance patterns

Our analysis highlights significant geographic variability in resistance patterns, with Iran, Pakistan, Türkiye and Iraq contributing the majority of studies. These countries reported high resistance rates among key pathogens, including *S. aureus, A. baumannii and K. pneumoniae*. *K. pneumoniae* and *E. coli* were most resistant to third-generation cephalosporins while carbapenem resistance was mainly isolated to *A. baumannii* and *P. aeruginosa.* The data underscore the critical challenge posed by MRSA, particularly in non-paediatric populations in Iran and Pakistan, with rates as high as 94% in non-paediatric studies from Iran. Notably, high resistance rates were found in *S. aureus* isolates in Iraq (100% resistant to methicillin). However, <30 isolates from Iraq for each of these pathogens were included, making the significance of this result less generalizable. Similarly, carbapenem resistance in *A. baumannii* was alarmingly high in Türkiye and Iran, reaching 95% and 82%, respectively.

### Resistance in sepsis, burns and wound infections

The resistance data for sepsis, burns and wound-related infections reveal concerning trends. In sepsis, Gram-negative pathogens such as *Sa. Typhi* and *E. coli* showed significant resistance, particularly in Türkiye and Pakistan. The high resistance rates to ciprofloxacin in *Sa. Typhi* in Pakistan (78%) suggest the need for alternative therapeutic strategies. Of interest, in patients with sepsis, when comparing studies reporting on non-paediatric patients and paediatric patients from the same country, rates of resistance were variable and not consistently higher in non-paediatric patients.

For burn infections, *P. aeruginosa* and *A. baumannii* were the predominant pathogens with high resistance rates, particularly in Iran and Iraq. Wound infections also demonstrated substantial resistance, with *S. aureus* being the most frequently reported pathogen across multiple countries.

### Limitations

This analysis has several limitations and delimitations. We used the JBI tool for prevalence studies (see ‘Methods’ section) to assess the quality of the included studies as the questions pertaining exposure, outcomes and confounding did not apply to the type of studies being included. In many studies, some key pieces of information were not specified, such as the target population (e.g. whether paediatric or adult, outpatient or inpatient) or the full methodology (e.g. retrospective versus prospective cross-sectional), limiting the amount of data available to classify the resistance types and pathogens. This limitation also prevented further analysis by population demographics. The methodological quality of studies overall varied, with papers from Yemen, Syria and Iran generally showing higher quality compared with those from Lebanon, Pakistan and Palestine. The lack of standardized methodologies and insufficient sample sizes in many studies highlight the need for more rigorous research designs and adherence to standardized guidelines. In some countries, only a single study on the infection site in question had been published (and met inclusion criteria) for the period of analysis, which both underscores the need for more and better surveillance and also limits our ability to draw strong conclusions from the information. Some papers were excluded based on concerns about their accuracy, completeness or the quality of the information (e.g. table information that did not match narrative text or insufficient description of quality control and methodology). The under-representation of certain countries and the limited number of isolates reported in some studies further restrict the generalizability of findings.

Regarding burns and wound/soft tissue/skin infection sites, the review includes papers that report on swab samples and therefore may include organisms not causative of the infection (i.e. contaminants). We also included studies that had >10 but <30 isolates, limiting the number of isolates analysed, which further limits the interpretation of the studies. However, we attempted to correct this limitation in the analysis by only including data for which there were at least 30 isolates, in combination. In addition, some of the studies reported resistance to certain antibiotics in species considered to be inherently resistant to them, i.e. ceftriaxone in *P. aeruginosa*, underscoring limitations in the quality of laboratory data and ABR reporting. Caution should be exercised when interpreting these results, especially for the purpose of updating empirical treatment, since (i) information about the number of isolates per patient was not available, (ii) information about hospitalization, as a proxy for severity of infection, was missing or combined (inpatient/outpatient) in 44% of patients included in the analysis, which may result in over or underestimation of resistance rates, (iii) information about the duration of hospitalization was also not included, therefore not permitting assessment of hospital-acquired versus community-acquired infection, as few papers reported this distinction and (iv) in many cases, the age cohort (i.e. paediatric or adult) of the patients was not reported.

Finally, the high reported resistance of Salmonella to ciprofloxacin in Pakistan is questionable and should be interpreted with caution considering the very low JBI score of this paper (1/8). Standard practice dictates that pefloxacin should be used to define ciprofloxacin resistance in Salmonella. If ciprofloxacin disc diffusion was used instead, this could explain the inflated resistance rates observed. However, although the data quality of this paper is low and the findings are questionable, considering the other reported papers on Salmonella in Pakistan (Figure 4), we do not expect these results to skew the overall interpretation of the findings. Similarly, the reported high resistance of Staphylococcus to vancomycin is problematic. Vancomycin resistance in Staphylococcus should be determined using minimum inhibitory concentration methods. The use of disc diffusion for vancomycin susceptibility testing could result in falsely elevated resistance rates. These methodological discrepancies underscore the need for standardized testing protocols to ensure the accuracy and reliability of ABR data. Additionally, the reliance on the Kirby–Bauer disc diffusion test, while common, may not capture the full spectrum of resistance mechanisms, suggesting a need for more advanced diagnostic techniques.

## Conclusion

In our meta-analysis, we analysed country-level antibiograms in nine countries of high clinical and operational priority to MSF and found varying levels of resistance, with high rates of MRSA and ESBL resistance. Despite multiple high reported rates of ABR across contexts, data quality limits the clinical usability of these data for individual patient care, with the exception that the BSI data may be useful to anticipate types and potentially quantities of antibiotics needed. The takeaway for MSF as a clinical provider is that these findings justify prioritizing some countries for more ABR control activities and also urgently underscore the need for advocacy at the national and international levels. It is particularly notable that though each of the included countries is enrolled in the GLASS system, the review still did not find better-quality data.

Nonetheless, the high resistance rates observed necessitate the urgent implementation of robust antimicrobial stewardship programmes in the studied regions. Clinicians should be guided by local resistance patterns when selecting empirical therapies, and there is a clear need for updated treatment guidelines that reflect current resistance data. The findings also underscore the importance of infection control measures to prevent the spread of resistant pathogens.

Future research should aim to address the methodological shortcomings identified in this review by employing more rigorous study designs, ensuring adequate sample sizes and adhering to standardized resistance definitions. Collaborative efforts between countries could facilitate the sharing of data and best practices, ultimately leading to more effective interventions. Additionally, research should explore the drivers of resistance in these regions, including antibiotic prescribing practices and infection control measures. Now is the time to harness the advancements in antibiotic stewardship and surveillance to make a tangible impact on the fight against antimicrobial resistance, ultimately ensuring better health outcomes for populations in LMICs.

## Supplementary Material

dlaf010_Supplementary_Data

## Supplementary data

Figures S1–S15 and Tables S1 and S2 are available as Supplementary data at *JAC-AMR* Online.
