# Supplementary material for: Antibiotic resistance in the Middle East and Southern Asia: a systematic review and meta-analysis
Source: JAC Antimicrob Resist. 2025 Feb 19;7(1):dlaf010. doi: 10.1093/jacamr/dlaf010 (PMC11836886; doi:10.1093/jacamr/dlaf010)
Supplement: dlaf010_Supplementary_Data [file dlaf010_supplementary_data.docx]

Supplementary materials

JAC-AMR-2024-314.R1

Manuscript title: “Antibiotic Resistance in the Middle East & Southern Asia: A Systematic Review and Meta-Analysis”

| **Table S1.** PRISMA Guidelines | | | |
| --- | --- | --- | --- |
| **Section and Topic** | **Item #** | **Checklist item** | **Location where item is reported** |
| **TITLE** | | |  |
| Title | 1 | Identify the report as a systematic review. | Title |
| **ABSTRACT** | | |  |
| Abstract | 2 | See the PRISMA 2020 for Abstracts checklist. | Abstract |
| **INTRODUCTION** | | |  |
| Rationale | 3 | Describe the rationale for the review in the context of existing knowledge. | Introduction, lines 69-94 |
| Objectives | 4 | Provide an explicit statement of the objective(s) or question(s) the review addresses. | Introduction, lines 95-100 |
| **METHODS** | | |  |
| Eligibility criteria | 5 | Specify the inclusion and exclusion criteria for the review and how studies were grouped for the syntheses. | Methods, section 2.1 |
| Information sources | 6 | Specify all databases, registers, websites, organisations, reference lists and other sources searched or consulted to identify studies. Specify the date when each source was last searched or consulted. | Methods, lines 110-112 |
| Search strategy | 7 | Present the full search strategies for all databases, registers and websites, including any filters and limits used. | Methods lines 110-121 |
| Selection process | 8 | Specify the methods used to decide whether a study met the inclusion criteria of the review, including how many reviewers screened each record and each report retrieved, whether they worked independently, and if applicable, details of automation tools used in the process. | Methods, lines 124-125 |
| Data collection process | 9 | Specify the methods used to collect data from reports, including how many reviewers collected data from each report, whether they worked independently, any processes for obtaining or confirming data from study investigators, and if applicable, details of automation tools used in the process. | Methods, line 125 |
| Data items | 10a | List and define all outcomes for which data were sought. Specify whether all results that were compatible with each outcome domain in each study were sought (e.g. for all measures, time points, analyses), and if not, the methods used to decide which results to collect. | Methods, lines 125-132 |
|  | 10b | List and define all other variables for which data were sought (e.g. participant and intervention characteristics, funding sources). Describe any assumptions made about any missing or unclear information. | Methods, line 135-152 |
| Study risk of bias assessment | 11 | Specify the methods used to assess risk of bias in the included studies, including details of the tool(s) used, how many reviewers assessed each study and whether they worked independently, and if applicable, details of automation tools used in the process. | Methods, Section 2.2 |
| Effect measures | 12 | Specify for each outcome the effect measure(s) (e.g. risk ratio, mean difference) used in the synthesis or presentation of results. | Methods, lines 164-173 |
| Synthesis methods | 13a | Describe the processes used to decide which studies were eligible for each synthesis (e.g. tabulating the study intervention characteristics and comparing against the planned groups for each synthesis (item #5)). | N/A |
|  | 13b | Describe any methods required to prepare the data for presentation or synthesis, such as handling of missing summary statistics, or data conversions. | Methods, lines 135-152 |
|  | 13c | Describe any methods used to tabulate or visually display results of individual studies and syntheses. | Methods, lines 167-169 |
|  | 13d | Describe any methods used to synthesize results and provide a rationale for the choice(s). If meta-analysis was performed, describe the model(s), method(s) to identify the presence and extent of statistical heterogeneity, and software package(s) used. | Methods, lines 171-173 |
|  | 13e | Describe any methods used to explore possible causes of heterogeneity among study results (e.g. subgroup analysis, meta-regression). | Methods, lines 169-170 |
|  | 13f | Describe any sensitivity analyses conducted to assess robustness of the synthesized results. | N/A |
| Reporting bias assessment | 14 | Describe any methods used to assess risk of bias due to missing results in a synthesis (arising from reporting biases). | Methods, Section 2.2 |
| Certainty assessment | 15 | Describe any methods used to assess certainty (or confidence) in the body of evidence for an outcome. | N/A |
| **RESULTS** | | |  |
| Study selection | 16a | Describe the results of the search and selection process, from the number of records identified in the search to the number of studies included in the review, ideally using a flow diagram. | Results, lines 177-180 |
|  | 16b | Cite studies that might appear to meet the inclusion criteria, but which were excluded, and explain why they were excluded. | N/A |
| Study characteristics | 17 | Cite each included study and present its characteristics. | Results, lines 181-187 |
| Risk of bias in studies | 18 | Present assessments of risk of bias for each included study. | Results, lines 188-196 |
| Results of individual studies | 19 | For all outcomes, present, for each study: (a) summary statistics for each group (where appropriate) and (b) an effect estimate and its precision (e.g. confidence/credible interval), ideally using structured tables or plots. | Table 1, Table 2, Figures 3-11, and Figures S1-S15 |
| Results of syntheses | 20a | For each synthesis, briefly summarise the characteristics and risk of bias among contributing studies. | Results, lines 188-196 |
|  | 20b | Present results of all statistical syntheses conducted. If meta-analysis was done, present for each the summary estimate and its precision (e.g. confidence/credible interval) and measures of statistical heterogeneity. If comparing groups, describe the direction of the effect. | Figures 3-11 and Tables S1-S15 |
|  | 20c | Present results of all investigations of possible causes of heterogeneity among study results. | N/A |
|  | 20d | Present results of all sensitivity analyses conducted to assess the robustness of the synthesized results. | N/A |
| Reporting biases | 21 | Present assessments of risk of bias due to missing results (arising from reporting biases) for each synthesis assessed. | N/A |
| Certainty of evidence | 22 | Present assessments of certainty (or confidence) in the body of evidence for each outcome assessed. | N/A |
| **DISCUSSION** | | |  |
| Discussion | 23a | Provide a general interpretation of the results in the context of other evidence. | N/A |
|  | 23b | Discuss any limitations of the evidence included in the review. | Discussion, Limitations, lines 403-445 |
|  | 23c | Discuss any limitations of the review processes used. | Discussion, lines 401-403 |
|  | 23d | Discuss implications of the results for practice, policy, and future research. | Conclusion |
| **OTHER INFORMATION** | | |  |
| Registration and protocol | 24a | Provide registration information for the review, including register name and registration number, or state that the review was not registered. | Declarations, lines 498-499 |
|  | 24b | Indicate where the review protocol can be accessed, or state that a protocol was not prepared. | N/A |
|  | 24c | Describe and explain any amendments to information provided at registration or in the protocol. | N/A |
| Support | 25 | Describe sources of financial or non-financial support for the review, and the role of the funders or sponsors in the review. | Declarations, lines 492-495 |
| Competing interests | 26 | Declare any competing interests of review authors. | Declarations, line 472 |
| Availability of data, code and other materials | 27 | Report which of the following are publicly available and where they can be found: template data collection forms; data extracted from included studies; data used for all analyses; analytic code; any other materials used in the review. | Declarations, line 497 |

| **Table S2.** Application of questions in the JBI Critical Appraisal tool to the included studies | | | | | | | | | | | | | |  |  |
| --- | --- | --- | --- | --- | --- | --- | --- | --- | --- | --- | --- | --- | --- | --- | --- |
|  | **Question** | | | | **Application to Included Studies** | | | | | | | | |  |  |
| 1 | Was the sample frame appropriate to address the target population? | | | | Any studies that limited their population to a narrow group were excluded from the analyses at the screening phase. Studies were marked “No” if they had other limiting factors based on characteristics such as age range, gender, morbidities, medications, etc. | | | | | | | | |  |  |
| 2 | Were study participants recruited in an appropriate way? | | | | Random probabilistic sampling did not apply to the type of studies included in this review. The appropriate recruitment of participants (aka samples) into the included studies is if only one pathogen was included per patient. If the papers did not explicitly state this, studies were marked as “Unclear”. | | | | | | | | |  |  |
| 3 | Was the sample size adequate? | | | | If the pathogens in the study had fewer than 30 isolates per pathogen per site, then this was marked as "No". If the number of isolates per pathogen per site was greater than 30 for all reported pathogens/sites of interest reported in the study, then this was marked "Yes". | | | | | | | | |  |  |
| 4 | Were the study subjects and setting described in detail? | | | | If the study did not state either the age range of the sample (e.g., 18+) or the setting (e.g., inpatient), then the response was "No". Both elements needed to be present to be marked “Yes”. | | | | | | | | |  |  |
| 5 | Was data analysis conducted with sufficient coverage of the identified sample? | | | | If for any reason a subset of the identified samples was left out of the final resistance analysis, this was marked “No”. | | | | | | | | |  |  |
| 6 | Were valid methods used for the identification of the condition? | | | | If studies did not state the method of resistance detection (e.g., disc diffusion), this was marked “No”. If a detailed description was provided, this was marked “Yes”. | | | | | | | | |  |  |
| 7 | Was the condition measured in a standard, reliable way for all participants? | | | | If the study methods mentioned the guidelines that were followed (e.g., CLSI), then this was marked “Yes”. If not, this was marked “No”. | | | | | | | | |  |  |
| 8 | Was there appropriate statistical analysis? | | | | The focus of this review is to identify rates of resistance, which is a fairly simple analysis. If calculations were unclear or found to be incorrect, then studies were marked “Unclear” or “No”, respectively. All studies with clearly calculated and presented results were marked “Yes”. | | | | | | | | |  |  |
| 9 | Was the response rate adequate, and if not, was the low response rate managed appropriately? | | | | This question was deemed *Not Applicable* since the majority of studies were cross-sectional from a deidentified laboratory/hospital database so patients were unable to dropout or refuse participation in the study. | | | | | | | | |  |  |
| Note: Each question in the JBI Critical Appraisal tool is scored in a binary manner, with “yes” leading to a score of 1, and “no” or “unclear” leading to a score of 0, for the respective question. The sum of the 9 individual question scores equates to the total score. In the case of our analysis, one question (Q9) was deemed not applicable so the score is out of a total of 8 questions. | | | | | | | | | | | | | |  |  |
|  | | | | | | | | | | | | | |  |  |
| **Figure S1.** Resistance patterns of *A. baumannii*, *E. coli*, *K. pneumoniae*, *P. aeruginosa, S. aureus,* and *S.* Typhi isolates found in blood stream infections (Sepsis) to the antimicrobial agents of interest in Iran – limited to studies reporting on the non-paediatric studies (N=10)**^14,16,18,19,22,24,26–29^** | | | | | | | | | | | | | | |  |
| **Antimicrobial Agent** | | **Amikacin** | **Carbapenem** | **Ceftriaxone** | | **Ciprofloxacin** | **Colistin** | **ESBL** | **Gentamicin** | **MRSA** | | **Vancomycin** | | |  |
|  |  | Proportion (95% CI)  (# isolates) | Proportion (95% CI)  (# isolates) | Proportion (95% CI)  (# isolates) | | Proportion (95% CI)  (# isolates) | Proportion (95% CI)  (# isolates) | Proportion (95% CI)  (# isolates) | Proportion (95% CI)  (# isolates) | Proportion (95% CI)  (# isolates) | | Proportion (95% CI)  (# isolates) | | |  |
| ***A. baumannii*** | |  |  |  | |  |  |  |  |  | |  | | |  |
|  | | - | 0.79  (73) | - | | - | - | - | - | - | | - | | |  |
| ***E. coli*** | |  |  |  | |  |  |  |  |  | |  | | |  |
|  | | 0.08 (0.04, 0.13)  (151) | 0.12 (0.07, 0.19)  (137) | 0.46 (0.38, 0.54)  (151) | | 0.53 (0.20, 0.84)  (165) | 0.17  (123) | 0.67  (123) | 0.24 (0.05, 0.50)  (165) | - | | - | | |  |
| ***K. pneumoniae*** | |  |  |  | |  |  |  |  |  | |  | | |  |
|  | | 0.08 (0.02, 0.18)  (51) | 0.06 (0.01, 0.16)  (51) | 0.50  (38) | | 0.49 (0.35, 0.63)  (51) | 0.16  (38) | 0.61  (38) | 0.31 (0.18, 0.44)  (51) | - | | - | | |  |
| ***P. aeruginosa*** | |  |  |  | |  |  |  |  |  | |  | | |  |
|  | | 0.53 (0.15, 0.89)  (165) | 0.32 (0.26, 0.39)  (197) | 0.42  (66) | | 0.70 (0.13, 1.00)  (165) | 0.08 (0.03, 0.14)  (99) | 0.45  (98) | 0.53 (0.22, 0.82)  (165) | - | | - | | |  |
| ***S. aureus*** | |  |  |  | |  |  |  |  |  | |  | | |  |
|  | | 0.27 (0.21, 0.34)  (199) | 0.36  (170) | 0.50 (0.43, 0.57)  (199) | | 0.28 (0.21, 0.34)  (199) | - | - | 0.33 (0.27, 0.40)  (199) | 0.94 (0.90, 0.97)  (209) | | 0.05  (170) | | |  |
| ***S.* Typhi** | |  |  |  | |  |  |  |  |  | |  | | |  |
|  | | - | - | - | | - | - | - | - | - | | - | | |  |
| When combining less than two studies, the biostatistical analysis was limited. Therefore, mean resistance without the 95% CI was reported. | | | | | | | | | | |  | |  | |  |
| *Acinetobacter baumannii* (*A. baumannii*), *Escherichia coli* (*E. coli*), extended-spectrum beta-lactamase (ESBL), *Klebsiella pneumoniae* (*K. pneumoniae*), methicillin-resistant *Staphylococcus aureus* (MRSA), *Pseudomonas aeruginosa* (*P. aeruginosa*), *Staphylococcus aureus* (*S. aureus*), *Salmonella* Typhi (*S*. Typhi) | | | | | | | | | | | | | | |  |
|  | | | | | | | | | | | | | | |  |
| **Legend** | | | | | | | | | | | | | | |  |
| < 20% Resistance | | | 20-39% Resistance | | | 40-59% Resistance | | 60-79% Resistance | | ≥ 80% Resistance | | | | |  |

| **Figure S2.** Resistance patterns of *A. baumannii*, *E. coli*, *K. pneumoniae*, *P. aeruginosa, S. aureus,* and *S.* Typhi isolates found in blood stream infections (Sepsis) to the antimicrobial agents of interest in Iran – limited to studies reporting on the paediatric studies (N=7)**^13,15,17,20,21,23,25^** | | | | | | | | | | | | | | |
| --- | --- | --- | --- | --- | --- | --- | --- | --- | --- | --- | --- | --- | --- | --- |
| **Antimicrobial Agent** | **Amikacin** | **Carbapenem** | | **Ceftriaxone** | **Ciprofloxacin** | | **Colistin** | | **ESBL** | **Gentamicin** | | **MRSA** | **Vancomycin** | |
|  | Proportion (95% CI)  (# isolates) | Proportion (95% CI)  (# isolates) | | Proportion (95% CI)  (# isolates) | Proportion (95% CI)  (# isolates) | | Proportion (95% CI)  (# isolates) | | Proportion (95% CI)  (# isolates) | Proportion (95% CI)  (# isolates) | | Proportion (95% CI)  (# isolates) | Proportion (95% CI)  (# isolates) | |
| ***A. baumannii*** |  |  | |  |  | |  | |  |  | |  |  | |
|  | 0.37 (0.29, 0.44)  (160) | 0.84  (86) | | 0.45  (74) | 0.59 (0.51, 0.66)  (160) | | - | | - | 0.50 (0.42, 0.58)  (160) | | - | - | |
| ***E. coli*** |  |  | |  |  | |  | |  |  | |  |  | |
|  | 0.20 (0.15, 0.26)  (260) | 0.06 (0.03, 0.10)  (223) | | 0.25 (0.01, 0.63)  (68) | 0.32 (0.26, 0.38)  (260) | | - | | - | 0.28 (0.19, 0.38)  (260) | | - | - | |
| ***K. pneumoniae*** |  |  | |  |  | |  | |  |  | |  |  | |
|  | 0.64  (263) | 0.18  (263) | | - | 0.00  (263) | | - | | - | 0.62  (263) | | - | - | |
| ***P. aeruginosa*** |  |  | |  |  | |  | |  |  | |  |  | |
|  | 0.29 (0.22, 0.38)  (128) | 0.21  (111) | | - | - | | - | | - | 0.25 (0.18, 0.33)  (128) | | - | - | |
| ***S. aureus*** |  |  | |  |  | |  | |  |  | |  |  | |
|  | 0.14 (0.00, 0.46)  (72) | 0.08 (0.02, 0.18)  (49) | | 0.29 (0.17, 0.44)  (47) | 0.27 (0.14, 0.43)  (110) | | - | | - | 0.26 (0.10, 0.44)  (110) | | 0.69 (0.57, 0.80)  (67) | 0.01 (0.00, 0.08)  (86) | |
| ***S.* Typhi** |  |  | |  |  | |  | |  |  | |  |  | |
|  | - | - | | - | - | | - | | - | - | | - | - | |
| When combining less than two studies, the biostatistical analysis was limited. Therefore, mean resistance without the 95% CI was reported. | | | | | | | | | | | | | |  |
| *Acinetobacter baumannii* (*A. baumannii*), *Escherichia coli* (*E. coli*), extended-spectrum beta-lactamase (ESBL), *Klebsiella pneumoniae* (*K. pneumoniae*), methicillin-resistant *Staphylococcus aureus* (MRSA), *Pseudomonas aeruginosa* (*P. aeruginosa*), *Staphylococcus aureus* (*S. aureus*), *Salmonella* Typhi (*S*. Typhi) | | | | | | | | | | | | | | |
|  | | | | | | | | | | | | | | |
| **Legend** | | | | | | | | | | | | | | |
| < 20% Resistance | | | 20-39% Resistance | | | 40-59% Resistance | | 60-79% Resistance | | | ≥ 80% Resistance | | | |

| **Figure S3.** Resistance patterns of *A. baumannii*, *E. coli*, *K. pneumoniae*, *P. aeruginosa, S. aureus, and S.* Typhi isolates found in blood stream infections (Sepsis) to the antimicrobial agents of interest in Pakistan – limited to studies reporting on the non-paediatric studies (N=20)**^35,37,42,43,45–48,50–54,57,58,60,61,64,236,242^** | | | | | | | | | |  |  |
| --- | --- | --- | --- | --- | --- | --- | --- | --- | --- | --- | --- |
| **Antimicrobial Agent** | **Amikacin** | **Carbapenem** | **Ceftriaxone** | **Ciprofloxacin** | **Colistin** | **ESBL** | **Gentamicin** | **MRSA** | **Vancomycin** |  |  |
|  | Proportion (95% CI)  (# isolates) | Proportion (95% CI)  (# isolates) | Proportion (95% CI)  (# isolates) | Proportion (95% CI)  (# isolates) | Proportion (95% CI)  (# isolates) | Proportion (95% CI)  (# isolates) | Proportion (95% CI)  (# isolates) | Proportion (95% CI)  (# isolates) | Proportion (95% CI)  (# isolates) |  |  |
| ***A. baumannii*** |  |  |  |  |  |  |  |  |  |  |  |
|  | 0.90  (156) | 0.90  (156) | 0.92  (156) | 0.81  (156) | - | - | 0.87  (156) | - | - |  |  |
| ***E. coli*** |  |  |  |  |  |  |  |  |  |  |  |
|  | 0.08 (0.04, 0.13)  (148) | 0.08 (0.05, 0.12)  (206) | 0.74 (0.66, 0.80)  (148) | 0.73 (0.65, 0.80)  (148) | 0.00 (0.00, 0.02)  (108) | 0.68 (0.53, 0.81)  (46) | 0.48  (98) | - | - |  |  |
| ***K. pneumoniae*** |  |  |  |  |  |  |  |  |  |  |  |
|  | 0.76  (71) | 0.75  (71) | 0.90  (71) | - | - | - | - | - | - |  |  |
| ***P. aeruginosa*** |  |  |  |  |  |  |  |  |  |  |  |
|  | 0.10  (67) | 0.10 (0.04, 0.18)  (82) | - | 0.46  (67) | 0.00 (0.00, 0.02)  (82) | - | - | - | - |  |  |
| ***S. aureus*** |  |  |  |  |  |  |  |  |  |  |  |
|  | 0.08  (73) | - | - | 0.33  (73) | - | - | - | 0.38 (0.28, 0.49)  (90) | 0.05 (0.01, 0.11)  (90) |  |  |
| ***S.* Typhi** |  |  |  |  |  |  |  |  |  |  |  |
|  | - | 0.00 (0.00, 0.03)  (1,076) | 0.12 (0.01, 0.32)  (10,168) | 0.83 (0.74, 0.90)  (12,028) | - | 0.02  (57) | - | - | - |  |  |
| When combining less than two studies, the biostatistical analysis was limited. Therefore, mean resistance without the 95% CI was reported. | | | | | | | | | |  |  |
| *Acinetobacter baumannii* (*A. baumannii*), *Escherichia coli* (*E. coli*), extended-spectrum beta-lactamase (ESBL), *Klebsiella pneumoniae* (*K. pneumoniae*), methicillin-resistant *Staphylococcus aureus* (MRSA), *Pseudomonas aeruginosa* (*P. aeruginosa*), *Staphylococcus aureus* (*S. aureus*), *Salmonella* Typhi (*S*. Typhi) | | | | | | | | | |  |  |
|  | | | | | | | | | |  |  |
| **Legend** | | | | | | | | | |  |  |
| < 20% Resistance | | 20-39% Resistance | | 40-59% Resistance | | 60-79% Resistance | | ≥ 80% Resistance | |  |  |

| **Figure S4.** Resistance patterns of *A. baumannii*, *E. coli*, *K. pneumoniae*, *P. aeruginosa, S. aureus, and S.* Typhi isolates found in blood stream infections (Sepsis) to the antimicrobial agents of interest in Pakistan – limited to studies reporting on the paediatric population (N=18)**^30–34,36,38–41,44,49,55,56,59,62,63,174^** | | | | | | | | | |  |
| --- | --- | --- | --- | --- | --- | --- | --- | --- | --- | --- |
| **Antimicrobial Agent** | **Amikacin** | **Carbapenem** | **Ceftriaxone** | **Ciprofloxacin** | **Colistin** | **ESBL** | **Gentamicin** | **MRSA** | **Vancomycin** |  |
|  | Proportion (95% CI)  (# isolates) | Proportion (95% CI)  (# isolates) | Proportion (95% CI)  (# isolates) | Proportion (95% CI)  (# isolates) | Proportion (95% CI)  (# isolates) | Proportion (95% CI)  (# isolates) | Proportion (95% CI)  (# isolates) | Proportion (95% CI)  (# isolates) | Proportion (95% CI)  (# isolates) |  |
| ***A. baumannii*** |  |  |  |  |  |  |  |  |  |  |
|  | 0.52 (0.00, 1.00)  (84) | 0.55 (0.02, 1.00)  (84) | 1.00 (0.95, 1.00)  (42) | 0.40 (0.00, 0.97)  (84) | 0.00 (0.00, 0.05)  (38) |  | 0.33 (0.00, 0.89)  (84) | - | - |  |
| ***E. coli*** |  |  |  |  |  |  |  |  |  |  |
|  | 0.26 (0.07, 0.50)  (906) | 0.14 (0.04, 0.28)  (932) | 0.67 (0.48, 0.85)  (885) | 0.53 (0.31, 0.75)  (914) | - | - | 0.58 (0.25, 0.87)  (854) | - | - |  |
| ***K. pneumoniae*** |  |  |  |  |  |  |  |  |  |  |
|  | 0.55 (0.29, 0.80)  (264) | 0.28 (0.10, 0.50)  (264) | 0.97 (0.92, 1.00)  (167) | 0.50 (0.31, 0.70)  (212) | 0.02 (0.00, 0.13)  (66) | 0.43 (0.30, 0.57)  (52) | 0.73 (0.48, 0.93)  (224) | - | - |  |
| ***P. aeruginosa*** |  |  |  |  |  |  |  |  |  |  |
|  | 0.14 (0.32, 0.49)  (132) | 0.57 (0.21, 0.89)  (159) | - | 0.74 (0.15, 1.00)  (159) | 0.42  (110) | - | 0.55  (110) | - | - |  |
| ***S. aureus*** |  |  |  |  |  |  |  |  |  |  |
|  | 0.30 (0.11, 0.55)  (714) | 0.43 (0.15, 0.74)  (612) | 0.65 (0.34, 0.91)  (452) | 0.56 (0.42, 0.69)  (1,094) | - | - | 0.63 (0.46, 0.79)  (766) | 0.62 (0.41, 0.82)  (391) | 0.19 (0.04, 0.42)  (1,071) |  |
| ***S.* Typhi** |  |  |  |  |  |  |  |  |  |  |
|  | 0.00  (415) | 0.13 (0.01, 0.34)  (714) | 0.33 (0.07, 0.66)  (299) | 0.59 (0.41, 0.76)  (714) | 0.00  (415) | 0.00  (35) | 0.00 (0.00, 0.00)  (426) | - | - |  |
| When combining less than two studies, the biostatistical analysis was limited. Therefore, mean resistance without the 95% CI was reported. | | | | | | | | | |  |
| *Acinetobacter baumannii* (*A. baumannii*), *Escherichia coli* (*E. coli*), extended-spectrum beta-lactamase (ESBL), *Klebsiella pneumoniae* (*K. pneumoniae*), methicillin-resistant *Staphylococcus aureus* (MRSA), *Pseudomonas aeruginosa* (*P. aeruginosa*), *Staphylococcus aureus* (*S. aureus*), *Salmonella* Typhi (*S*. Typhi) | | | | | | | | | |  |
|  | | | | | | | | | |  |
| **Legend** | | | | | | | | | |  |
| < 20% Resistance | | 20-39% Resistance | | 40-59% Resistance | | 60-79% Resistance | | ≥ 80% Resistance | |  |

| **Figure S5.** Resistance patterns of *A. baumannii*, *E. coli*, *K. pneumoniae*, *P. aeruginosa, S. aureus, and S.* Typhi isolates found in blood stream infections (Sepsis) to the antimicrobial agents of interest in Türkiye – limited to studies reporting on the non-paediatric population (N=22)**^65,67–71,73,75,77–82,84–86,152,157,166,179,181^** | | | | | | | | | |  |
| --- | --- | --- | --- | --- | --- | --- | --- | --- | --- | --- |
| **Antimicrobial Agent** | **Amikacin** | **Carbapenem** | **Ceftriaxone** | **Ciprofloxacin** | **Colistin** | **ESBL** | **Gentamicin** | **MRSA** | **Vancomycin** |  |
|  | Proportion (95% CI)  (# isolates) | Proportion (95% CI)  (# isolates) | Proportion (95% CI)  (# isolates) | Proportion (95% CI)  (# isolates) | Proportion (95% CI)  (# isolates) | Proportion (95% CI)  (# isolates) | Proportion (95% CI)  (# isolates) | Proportion (95% CI)  (# isolates) | Proportion (95% CI)  (# isolates) |  |
| ***A. baumannii*** |  |  |  |  |  |  |  |  |  |  |
|  | 0.61 (0.44, 0.77)  (500) | 0.95 (0.92, 0.97)  (1,193) | 0.97  (256) | 0.95 (0.92, 0.97)  (1,193) | 0.01 (0.00, 0.03)  (946) | - | 0.61 (0.50, 0.72)  (1,173) | - | - |  |
| ***E. coli*** |  |  |  |  |  |  |  |  |  |  |
|  | 0.10 (0.04, 0.18)  (625) | 0.04 (0.00, 0.10)  (1,919) | 0.57 (0.50, 0.65)  (534) | 0.53 (0.46, 0.60)  (1,162) | 0.02 (0.00, 0.08)  (484) | 0.48 (0.40, 0.56)  (381) | 0.27 (0.24, 0.30)  (822) | - | - |  |
| ***K. pneumoniae*** |  |  |  |  |  |  |  |  |  |  |
|  | 0.45 (0.29, 0.62)  (679) | 0.40 (0.30, 0.50)  (2,061) | 0.75 (0.64, 0.85)  (634) | 0.56 (0.47, 0.66)  (1,316) | 0.19 (0.05, 0.40)  (1,229) | 0.24  (59) | 0.49 (0.38, 0.61)  (1,257) | - | - |  |
| ***P. aeruginosa*** |  |  |  |  |  |  |  |  |  |  |
|  | 0.19 (0.11, 0.29)  (255) | 0.44 (0.30, 0.58)  (534) | 0.51  (74) | 0.40 (0.29, 0.51)  (534) | 0.05 (0.02, 0.09)  (409) | - | 0.23 (0.15, 0.32)  (483) | - | - |  |
| ***S. aureus*** |  |  |  |  |  |  |  |  |  |  |
|  | - | - | - | 0.40 (0.17, 0.65)  (254) | - | - | 0.46 (0.38, 0.53)  (2,270) | 0.58 (0.52, 0.64)  (2,413) | - |  |
| ***S.* Typhi** |  |  |  |  |  |  |  |  |  |  |
|  | - | - | - | - | - | - | - | - | - |  |
| When combining less than two studies, the biostatistical analysis was limited. Therefore, mean resistance without the 95% CI was reported. | | | | | | | | | |  |
| *Acinetobacter baumannii* (*A. baumannii*), *Escherichia coli* (*E. coli*), extended-spectrum beta-lactamase (ESBL), *Klebsiella pneumoniae* (*K. pneumoniae*), methicillin-resistant *Staphylococcus aureus* (MRSA), *Pseudomonas aeruginosa* (*P. aeruginosa*), *Staphylococcus aureus* (*S. aureus*), *Salmonella* Typhi (*S*. Typhi) | | | | | | | | | |  |
|  | | | | | | | | | |  |
| **Legend** | | | | | | | | | |  |
| < 20% Resistance | | 20-39% Resistance | | 40-59% Resistance | | 60-79% Resistance | | ≥ 80% Resistance | |  |

| **Figure S6.** Resistance patterns of *A. baumannii*, *E. coli*, *K. pneumoniae*, *P. aeruginosa, S. aureus, and S. T*yphi isolates found in blood stream infections (Sepsis) to the antimicrobial agents of interest in Türkiye – limited to studies reporting on the paediatric population (N=5)**^72,74,76,83,155^** | | | | | | | | | |  |  |
| --- | --- | --- | --- | --- | --- | --- | --- | --- | --- | --- | --- |
| **Antimicrobial Agent** | **Amikacin** | **Carbapenem** | **Ceftriaxone** | **Ciprofloxacin** | **Colistin** | **ESBL** | **Gentamicin** | **MRSA** | **Vancomycin** |  |  |
|  | Proportion (95% CI)  (# isolates) | Proportion (95% CI)  (# isolates) | Proportion (95% CI)  (# isolates) | Proportion (95% CI)  (# isolates) | Proportion (95% CI)  (# isolates) | Proportion (95% CI)  (# isolates) | Proportion (95% CI)  (# isolates) | Proportion (95% CI)  (# isolates) | Proportion (95% CI)  (# isolates) |  |  |
| ***A. baumannii*** |  |  |  |  |  |  |  |  |  |  |  |
|  | - | - | - | - | - | - | - | - | - |  |  |
| ***E. coli*** |  |  |  |  |  |  |  |  |  |  |  |
|  | 0.15 (0.08, 0.24)  (85) | 0.08 (0.00, 0.22)  (113) | 0.52  (60) | 0.40 (0.29, 0.51)  (85) | 0.03  (60) | 0.35  (57) | 0.26 (0.17, 0.36)  (85) | - | - |  |  |
| ***K. pneumoniae*** |  |  |  |  |  |  |  |  |  |  |  |
|  | 0.42  (114) | 0.23 (0.17, 0.28)  (225) | 0.71  (114) | 0.36  (114) | 0.05  (114) | 0.60 (0.53, 0.67)  (188) | 0.45  (114) | - | - |  |  |
| ***P. aeruginosa*** |  |  |  |  |  |  |  |  |  |  |  |
|  | - | 0.33  (39) | - | - | - | - | - | - | - |  |  |
| ***S. aureus*** |  |  |  |  |  |  |  |  |  |  |  |
|  | - | - | - | - | - | - | - | 0.44  (148) | - |  |  |
| ***S.* Typhi** |  |  |  |  |  |  |  |  |  |  |  |
|  | - | - | - | - | - | - | - | - | - |  |  |
| When combining less than two studies, the biostatistical analysis was limited. Therefore, mean resistance without the 95% CI was reported. | | | | | | | | | |  |  |
| *Acinetobacter baumannii* (*A. baumannii*), *Escherichia coli* (*E. coli*), extended-spectrum beta-lactamase (ESBL), *Klebsiella pneumoniae* (*K. pneumoniae*), methicillin-resistant *Staphylococcus aureus* (MRSA), *Pseudomonas aeruginosa* (*P. aeruginosa*), *Staphylococcus aureus* (*S. aureus*), *Salmonella* Typhi (*S*. Typhi) | | | | | | | | | |  |  |
|  | | | | | | | | | |  |  |
| **Legend** | | | | | | | | | |  |  |
| < 20% Resistance | | 20-39% Resistance | | 40-59% Resistance | | 60-79% Resistance | | ≥ 80% Resistance | |  |  |

| **Figure S7.** Resistance patterns of *A. baumannii*, *E. coli*, *K. pneumoniae*, *P. aeruginosa, S. aureus, and S.* Typhi isolates found in blood stream infections (Sepsis) to the antimicrobial agents of interest in Afghanistan**^88^** | | | | | | | | | | | | | |  |
| --- | --- | --- | --- | --- | --- | --- | --- | --- | --- | --- | --- | --- | --- | --- |
| **Antimicrobial Agent** | **Amikacin** | **Carbapenem** | | **Ceftriaxone** | **Ciprofloxacin** | | **Colistin** | | **ESBL** | **Gentamicin** | | **MRSA** | **Vancomycin** |  |
|  | Proportion (95% CI)  (# isolates) | Proportion (95% CI)  (# isolates) | | Proportion (95% CI)  (# isolates) | Proportion (95% CI)  (# isolates) | | Proportion (95% CI)  (# isolates) | | Proportion (95% CI)  (# isolates) | Proportion (95% CI)  (# isolates) | | Proportion (95% CI)  (# isolates) | Proportion (95% CI)  (# isolates) |  |
| ***A. baumannii*** |  |  | |  |  | |  | |  |  | |  |  |  |
|  | - | - | | - | - | | - | | - | - | | - | - |  |
| ***E. coli*** |  |  | |  |  | |  | |  |  | |  |  |  |
|  | - | - | | - | - | | - | | - | - | | - | - |  |
| ***K. pneumoniae*** |  |  | |  |  | |  | |  |  | |  |  |  |
|  | - | - | | - | - | | - | | - | - | | - | - |  |
| ***P. aeruginosa*** |  |  | |  |  | |  | |  |  | |  |  |  |
|  | - | - | | - | - | | - | | - | - | | - | - |  |
| ***S. aureus*** |  |  | |  |  | |  | |  |  | |  |  |  |
|  | - | - | | - | - | | - | | - | - | | 0.65  (270) | - |  |
| ***S.* Typhi** |  |  | |  |  | |  | |  |  | |  |  |  |
|  | - | - | | - | - | | - | | - | - | | - | - |  |
| When combining less than two studies, the biostatistical analysis was limited. Therefore, mean resistance without the 95% CI was reported. | | | | | | | | | | | | | |  |
| *Acinetobacter baumannii* (*A. baumannii*), *Escherichia coli* (*E. coli*), extended-spectrum beta-lactamase (ESBL), *Klebsiella pneumoniae* (*K. pneumoniae*), methicillin-resistant *Staphylococcus aureus* (MRSA), *Pseudomonas aeruginosa* (*P. aeruginosa*), *Staphylococcus aureus* (*S. aureus*), *Salmonella* Typhi (*S*. Typhi) | | | | | | | | | | | | | |  |
|  | | | | | | | | | | | | | |  |
| **Legend** | | | | | | | | | | | | | |  |
| < 20% Resistance | | | 20-39% Resistance | | | 40-59% Resistance | | 60-79% Resistance | | | ≥ 80% Resistance | | |  |

| **Figure S8.** Resistance patterns of *A. baumannii*, *E. coli*, *K. pneumoniae*, *P. aeruginosa, S. aureus, and S.* Typhi isolates found in blood stream infections (Sepsis) to the antimicrobial agents of interest in Iraq**^57,210^** | | | | | | | | | | |  |  |
| --- | --- | --- | --- | --- | --- | --- | --- | --- | --- | --- | --- | --- |
| **Antimicrobial Agent** | **Amikacin** | **Carbapenem** | **Ceftriaxone** | **Ciprofloxacin** | **Colistin** | **ESBL** | **Gentamicin** | **MRSA** | **Vancomycin** | |  |  |
|  | Proportion (95% CI)  (# isolates) | Proportion (95% CI)  (# isolates) | Proportion (95% CI)  (# isolates) | Proportion (95% CI)  (# isolates) | Proportion (95% CI)  (# isolates) | Proportion (95% CI)  (# isolates) | Proportion (95% CI)  (# isolates) | Proportion (95% CI)  (# isolates) | Proportion (95% CI)  (# isolates) | |  |  |
| ***A. baumannii*** |  |  |  |  |  |  |  |  |  | |  |  |
|  | - | - | - | - | - | - | - | - | - | |  |  |
| ***E. coli*** |  |  |  |  |  |  |  |  |  | |  |  |
|  | - | - | - | - | - | - | - | - | - | |  |  |
| ***K. pneumoniae*** |  |  |  |  |  |  |  |  |  | |  |  |
|  | - | - | - | - | - | - | - | - | - | |  |  |
| ***P. aeruginosa*** |  |  |  |  |  |  |  |  |  | |  |  |
|  | - | - | - | - | - | - | - | - | - | |  |  |
| ***S. aureus*** |  |  |  |  |  |  |  |  |  | |  |  |
|  | - | - | - | - | - | - | - | - | - | |  |  |
| ***S.* Typhi** |  |  |  |  |  |  |  |  |  | |  |  |
|  | - | - | 0.00  (59) | 0.72 (0.61, 0.82)  (74) | - | - | - | - | - | |  |  |
| When combining less than two studies, the biostatistical analysis was limited. Therefore, mean resistance without the 95% CI was reported. | | | | | | | | | | |  |  |
| *Acinetobacter baumannii* (*A. baumannii*), *Escherichia coli* (*E. coli*), extended-spectrum beta-lactamase (ESBL), *Klebsiella pneumoniae* (*K. pneumoniae*), methicillin-resistant *Staphylococcus aureus* (MRSA), *Pseudomonas aeruginosa* (*P. aeruginosa*), *Staphylococcus aureus* (*S. aureus*), *Salmonella* Typhi (*S*. Typhi) | | | | | | | | | | |  |  |
|  | | | | | | | | | | |  |  |
| **Legend** | | | | | | | | | |  |  |  |
| < 20% Resistance | | 20-39% Resistance | | 40-59% Resistance | | 60-79% Resistance | | ≥ 80% Resistance | | |  |  |

| **Figure S9.** Resistance patterns of  *A. baumannii*, *E. coli*, *K. pneumoniae*, *P. aeruginosa, S. aureus, and S.* Typhi isolates found in blood stream infections (Sepsis) to the antimicrobial agents of interest in Lebanon**^87,89^** | | | | | | | | | |  | |  |  |
| --- | --- | --- | --- | --- | --- | --- | --- | --- | --- | --- | --- | --- | --- |
| **Antimicrobial Agent** | **Amikacin** | **Carbapenem** | **Ceftriaxone** | **Ciprofloxacin** | **Colistin** | **ESBL** | **Gentamicin** | **MRSA** | **Vancomycin** |  | |  |  |
|  | Proportion (95% CI)  (# isolates) | Proportion (95% CI)  (# isolates) | Proportion (95% CI)  (# isolates) | Proportion (95% CI)  (# isolates) | Proportion (95% CI)  (# isolates) | Proportion (95% CI)  (# isolates) | Proportion (95% CI)  (# isolates) | Proportion (95% CI)  (# isolates) | Proportion (95% CI)  (# isolates) |  | |  |  |
| ***A. baumannii*** |  |  |  |  |  |  |  |  |  |  | |  |  |
|  | 0.90  (90) | 0.91  (90) | - | 0.96  (90) | 0.00  (90) | - | - | - | - |  | |  |  |
| ***E. coli*** |  |  |  |  |  |  |  |  |  |  | |  |  |
|  | - | - | - | - | - | - | - | - | - |  | |  |  |
| ***K. pneumoniae*** |  |  |  |  |  |  |  |  |  |  | |  |  |
|  | - | - | - | - | - | - | - | - | - |  | |  |  |
| ***P. aeruginosa*** |  |  |  |  |  |  |  |  |  |  | |  |  |
|  | - | - | - | - | - | - | - | - | - |  | |  |  |
| ***S. aureus*** |  |  |  |  |  |  |  |  |  |  | |  |  |
|  | - | - | - | - | - | - | - | 0.45 (0.38, 0.52)  (193) | - |  | |  |  |
| ***S.* Typhi** |  |  |  |  |  |  |  |  |  |  | |  |  |
|  | - | - | - | - | - | - | - | - | - |  | |  |  |
| When combining less than two studies, the biostatistical analysis was limited. Therefore, mean resistance without the 95% CI was reported. | | | | | | | | | | |  |  |  |
| *Acinetobacter baumannii* (*A. baumannii*), *Escherichia coli* (*E. coli*), extended-spectrum beta-lactamase (ESBL), *Klebsiella pneumoniae* (*K. pneumoniae*), methicillin-resistant *Staphylococcus aureus* (MRSA), *Pseudomonas aeruginosa* (*P. aeruginosa*), *Staphylococcus aureus* (*S. aureus*), *Salmonella* Typhi (*S*. Typhi) | | | | | | | | | | |  |  |  |
|  | | | | | | | | | | |  |  |  |
| **Legend** | | | | | | | | | | |  |  | |
| < 20% Resistance | | 20-39% Resistance | | 40-59% Resistance | | 60-79% Resistance | | ≥ 80% Resistance | | |  |  |  |

| **Figure S10.** Resistance patterns of *A. baumannii*, *E. coli*, *K. pneumoniae*, *P. aeruginosa, S. aureus, and S. pneumoniae* isolates found in burn-related infections to the antimicrobial agents of interest in Yemen**^118^** | | | | | | | | | |  |  | |  |
| --- | --- | --- | --- | --- | --- | --- | --- | --- | --- | --- | --- | --- | --- |
| **Antimicrobial Agent** | **Amikacin** | **Carbapenem** | **Ceftriaxone** | **Ciprofloxacin** | **Colistin** | **ESBL** | **Gentamicin** | **MRSA** | **Vancomycin** |  |  |  |  |
|  | Proportion (95% CI)  (# isolates) | Proportion (95% CI)  (# isolates) | Proportion (95% CI)  (# isolates) | Proportion (95% CI)  (# isolates) | Proportion (95% CI)  (# isolates) | Proportion (95% CI)  (# isolates) | Proportion (95% CI)  (# isolates) | Proportion (95% CI)  (# isolates) | Proportion (95% CI)  (# isolates) |  |  |  |  |
| ***A. baumannii*** |  |  |  |  |  |  |  |  |  |  |  |  |  |
|  | - | - | - | - | - | - | - | - | - |  |  |  |  |
| ***E. coli*** |  |  |  |  |  |  |  |  |  |  |  |  |  |
|  | - | - | - | - | - | - | - | - | - |  |  |  |  |
| ***K. pneumoniae*** |  |  |  |  |  |  |  |  |  |  |  |  |  |
|  | - | - | - | - | - | - | - | - | - |  |  |  |  |
| ***P. aeruginosa*** |  |  |  |  |  |  |  |  |  |  |  |  |  |
|  | 0.83  (46) | 0.22  (46) | - | 0.65  (46) | - | - | 0.87  (46) | - | - |  |  |  |  |
| ***S. aureus*** |  |  |  |  |  |  |  |  |  |  |  |  |  |
|  | - | - | - | - | - | - | - | - | - |  |  |  |  |
| ***S. pneumoniae*** |  |  |  |  |  |  |  |  |  |  |  |  |  |
|  | - | - | - | - | - | - | - | - | - |  |  |  |  |
| When combining less than two studies, the biostatistical analysis was limited. Therefore, mean resistance without the 95% CI was reported. | | | | | | | | | |  | |  | |
| *Acinetobacter baumannii* (*A. baumannii*), *Escherichia coli* (*E. coli*), extended-spectrum beta-lactamase (ESBL), *Klebsiella pneumoniae* (*K. pneumoniae*), methicillin-resistant *Staphylococcus aureus* (MRSA), *Pseudomonas aeruginosa* (*P. aeruginosa*), *Staphylococcus aureus* (S. aureus), *Streptococcus pneumoniae* (*S. pneumoniae*) | | | | | | | | | |  | |  | |
|  | | | | | | | | | |  | |  | |
| **Legend** | | | | | | | | | |  | |  | |
| < 20% Resistance | | 20-39% Resistance | | 40-59% Resistance | | 60-79% Resistance | | ≥ 80% Resistance | |  |  |  |  |

| **Figure S11.** Resistance patterns of *A. baumannii*, *E. coli*, *K. pneumoniae*, *P. aeruginosa, S. aureus, and S. epidermidis* isolates found in wound-related infections to the antimicrobial agents of interest in Iran**^20,22,134–141,147^** | | | | | | | | | |  |
| --- | --- | --- | --- | --- | --- | --- | --- | --- | --- | --- |
| **Antimicrobial Agent** | **Amikacin** | **Carbapenem** | **Ceftriaxone** | **Ciprofloxacin** | **Colistin** | **ESBL** | **Gentamicin** | **MRSA** | **Vancomycin** |  |
|  | Proportion (95% CI)  (# isolates) | Proportion (95% CI)  (# isolates) | Proportion (95% CI)  (# isolates) | Proportion (95% CI)  (# isolates) | Proportion (95% CI)  (# isolates) | Proportion (95% CI)  (# isolates) | Proportion (95% CI)  (# isolates) | Proportion (95% CI)  (# isolates) | Proportion (95% CI)  (# isolates) |  |
| ***A. baumannii*** |  |  |  |  |  |  |  |  |  |  |
|  | - | - | - | - | - | - | - | - | - |  |
| ***E. coli*** |  |  |  |  |  |  |  |  |  |  |
|  | - | - | - | - | - | - | - | - | - |  |
| ***K. pneumoniae*** |  |  |  |  |  |  |  |  |  |  |
|  | - | - | - | - | - | - | - | - | - |  |
| ***P. aeruginosa*** |  |  |  |  |  |  |  |  |  |  |
|  | - | - | - | - | - | - | - | - | - |  |
| ***S. aureus*** |  |  |  |  |  |  |  |  |  |  |
|  | 0.07  (30) | 0.07  (41) | 1.00  (41) | 0.32 (0.15, 0.52)  (292) | - | - | 0.21 (0.13, 0.31)  (89) | 0.65 (0.52, 0.76)  (620) | 0.00 (0.00, 0.01)  (172) |  |
| ***S. epidermidis*** |  |  |  |  |  |  |  |  |  |  |
|  | - | - | - | - | - | - | - | - | - |  |
| When combining less than two studies, the biostatistical analysis was limited. Therefore, mean resistance without the 95% CI was reported. | | | | | | | | | |  |
| *Acinetobacter baumannii* (*A. baumannii*), *Escherichia coli* (*E. coli*), extended-spectrum beta-lactamase (ESBL), *Klebsiella pneumoniae* (*K. pneumoniae*), methicillin-resistant *Staphylococcus aureus* (MRSA), *Pseudomonas aeruginosa* (*P. aeruginosa*), *Staphylococcus aureus* (*S. aureus*), *Staphylococcus epidermidis* (*S. epidermidis*) | | | | | | | | | |  |
|  | | | | | | | | | |  |
| **Legend** | | | | | | | | | |  |
| < 20% Resistance | | 20-39% Resistance | | 40-59% Resistance | | 60-79% Resistance | | ≥ 80% Resistance | |  |

| **Figure S12.** Resistance patterns of *A. baumannii*, *E. coli*, *K. pneumoniae*, *P. aeruginosa, S. aureus, and S. epidermidis* isolates found in wound-related infections to the antimicrobial agents of interest in Iraq**^133,149–151^** | | | | | | | | | |  |  | |  |
| --- | --- | --- | --- | --- | --- | --- | --- | --- | --- | --- | --- | --- | --- |
| **Antimicrobial Agent** | **Amikacin** | **Carbapenem** | **Ceftriaxone** | **Ciprofloxacin** | **Colistin** | **ESBL** | **Gentamicin** | **MRSA** | **Vancomycin** |  |  |  |  |
|  | Proportion (95% CI)  (# isolates) | Proportion (95% CI)  (# isolates) | Proportion (95% CI)  (# isolates) | Proportion (95% CI)  (# isolates) | Proportion (95% CI)  (# isolates) | Proportion (95% CI)  (# isolates) | Proportion (95% CI)  (# isolates) | Proportion (95% CI)  (# isolates) | Proportion (95% CI)  (# isolates) |  |  |  |  |
| ***A. baumannii*** |  |  |  |  |  |  |  |  |  |  |  |  |  |
|  | - | - | - | - | - | - | - | - | - |  |  |  |  |
| ***E. coli*** |  |  |  |  |  |  |  |  |  |  |  |  |  |
|  | - | - | - | - | - | - | - | - | - |  |  |  |  |
| ***K. pneumoniae*** |  |  |  |  |  |  |  |  |  |  |  |  |  |
|  | - | - | - | - | - | - | - | - | - |  |  |  |  |
| ***P. aeruginosa*** |  |  |  |  |  |  |  |  |  |  |  |  |  |
|  | - | - | - | - | - | - | - | - | - |  |  |  |  |
| ***S. aureus*** |  |  |  |  |  |  |  |  |  |  |  |  |  |
|  | - | 0.00  (32) | - | 0.06  (85) | - | - | 0.06  (32) | 0.36 (0.08, 0.71)  (255) | 0.00 (0.00, 0.02)  (178) |  |  |  |  |
| ***S. epidermidis*** |  |  |  |  |  |  |  |  |  |  |  |  |  |
|  | - | - | - | - | - | - | - | - | - |  |  |  |  |
| When combining less than two studies, the biostatistical analysis was limited. Therefore, mean resistance without the 95% CI was reported. | | | | | | | | | |  | |  | |
| *Acinetobacter baumannii* (*A. baumannii*), *Escherichia coli* (*E. coli*), extended-spectrum beta-lactamase (ESBL), *Klebsiella pneumoniae* (*K. pneumoniae*), methicillin-resistant *Staphylococcus aureus* (MRSA), *Pseudomonas aeruginosa* (*P. aeruginosa*), *Staphylococcus aureus* (*S. aureus*), *Staphylococcus epidermidis* (*S. epidermidis*) | | | | | | | | | |  | |  | |
|  | | | | | | | | | |  | |  | |
| **Legend** | | | | | | | | | |  | |  | |
| < 20% Resistance | | 20-39% Resistance | | 40-59% Resistance | | 60-79% Resistance | | ≥ 80% Resistance | |  | |  |  |

| **Figure S13.** Resistance patterns of *A. baumannii*, *E. coli*, *K. pneumoniae*, *P. aeruginosa, S. aureus, and S. epidermidis* isolates found in wound-related infections to the antimicrobial agents of interest in Syria**^132,133^** | | | | | | | | | |  |
| --- | --- | --- | --- | --- | --- | --- | --- | --- | --- | --- |
| **Antimicrobial Agent** | **Amikacin** | **Carbapenem** | **Ceftriaxone** | **Ciprofloxacin** | **Colistin** | **ESBL** | **Gentamicin** | **MRSA** | **Vancomycin** |  |
|  | Proportion (95% CI)  (# isolates) | Proportion (95% CI)  (# isolates) | Proportion (95% CI)  (# isolates) | Proportion (95% CI)  (# isolates) | Proportion (95% CI)  (# isolates) | Proportion (95% CI)  (# isolates) | Proportion (95% CI)  (# isolates) | Proportion (95% CI)  (# isolates) | Proportion (95% CI)  (# isolates) |  |
| ***A. baumannii*** |  |  |  |  |  |  |  |  |  |  |
|  | - | - | - | - | - | - | - | - | - |  |
| ***E. coli*** |  |  |  |  |  |  |  |  |  |  |
|  | - | - | - | - | - | - | - | - | - |  |
| ***K. pneumoniae*** |  |  |  |  |  |  |  |  |  |  |
|  | - | - | - | - | - | - | - | - | - |  |
| ***P. aeruginosa*** |  |  |  |  |  |  |  |  |  |  |
|  | - | - | - | - | - | - | - | - | - |  |
| ***S. aureus*** |  |  |  |  |  |  |  |  |  |  |
|  | - | - | - | - | - | - | - | 0.59 (0.45, 0.73)  (51) | - |  |
| ***S. epidermidis*** |  |  |  |  |  |  |  |  |  |  |
|  | - | - | - | - | - | - | - | - | - |  |
| When combining less than two studies, the biostatistical analysis was limited. Therefore, mean resistance without the 95% CI was reported. | | | | | | | | | |  |
| *Acinetobacter baumannii* (*A. baumannii*), *Escherichia coli* (*E. coli*), extended-spectrum beta-lactamase (ESBL), *Klebsiella pneumoniae* (*K. pneumoniae*), methicillin-resistant *Staphylococcus aureus* (MRSA), *Pseudomonas aeruginosa* (*P. aeruginosa*), *Staphylococcus aureus* (*S. aureus*), *Staphylococcus epidermidis* (*S. epidermidis*) | | | | | | | | | |  |
|  | | | | | | | | | |  |
| **Legend** | | | | | | | | | |  |
| < 20% Resistance | | 20-39% Resistance | | 40-59% Resistance | | 60-79% Resistance | | ≥ 80% Resistance | |  |

| **Figure S14.** Resistance patterns of *A. baumannii*, *E. coli*, *K. pneumoniae*, *P. aeruginosa, S. aureus, and S. epidermidis* isolates found in wound-related infections to the antimicrobial agents of interest in Türkiye**^142–146,148,191^** | | | | | | | | | |  |
| --- | --- | --- | --- | --- | --- | --- | --- | --- | --- | --- |
| **Antimicrobial Agent** | **Amikacin** | **Carbapenem** | **Ceftriaxone** | **Ciprofloxacin** | **Colistin** | **ESBL** | **Gentamicin** | **MRSA** | **Vancomycin** |  |
|  | Proportion (95% CI)  (# isolates) | Proportion (95% CI)  (# isolates) | Proportion (95% CI)  (# isolates) | Proportion (95% CI)  (# isolates) | Proportion (95% CI)  (# isolates) | Proportion (95% CI)  (# isolates) | Proportion (95% CI)  (# isolates) | Proportion (95% CI)  (# isolates) | Proportion (95% CI)  (# isolates) |  |
| ***A. baumannii*** |  |  |  |  |  |  |  |  |  |  |
|  | - | - | - | - | - | - | - | - | - |  |
| ***E. coli*** |  |  |  |  |  |  |  |  |  |  |
|  | - | - | - | - | - | - | - | - | - |  |
| ***K. pneumoniae*** |  |  |  |  |  |  |  |  |  |  |
|  | - | - | - | - | - | - | - | - | - |  |
| ***P. aeruginosa*** |  |  |  |  |  |  |  |  |  |  |
|  | - | - | - | - | - | - | - | - | - |  |
| ***S. aureus*** |  |  |  |  |  |  |  |  |  |  |
|  | 0.55  (69) | - | - | 0.01 (0.00, 0.04)  (142) | - | - | 0.05 (0.00, 0.38)  (384) | 0.25 (0.11, 0.42)  (557) | 0.00 (0.00, 0.01)  (142) |  |
| ***S. epidermidis*** |  |  |  |  |  |  |  |  |  |  |
|  | - | - | - | - | - | - | - | - | - |  |
| When combining less than two studies, the biostatistical analysis was limited. Therefore, mean resistance without the 95% CI was reported. | | | | | | | | | |  |
| *Acinetobacter baumannii* (*A. baumannii*), *Escherichia coli* (*E. coli*), extended-spectrum beta-lactamase (ESBL), *Klebsiella pneumoniae* (*K. pneumoniae*), methicillin-resistant *Staphylococcus aureus* (MRSA), *Pseudomonas aeruginosa* (*P. aeruginosa*), *Staphylococcus aureus* (*S. aureus*), *Staphylococcus epidermidis* (*S. epidermidis*) | | | | | | | | | |  |
|  | | | | | | | | | |  |
| **Legend** | | | | | | | | | |  |
| < 20% Resistance | | 20-39% Resistance | | 40-59% Resistance | | 60-79% Resistance | | ≥ 80% Resistance | |  |

| **Figure S15.** Resistance patterns of *A. baumannii*, *E. coli*, *K. pneumoniae*, *P. aeruginosa, S. aureus, and S. epidermidis* isolates found in wound-related infections to the antimicrobial agents of interest in Yemen**^118,131,133^** | | | | | | | | | | | | | |  |
| --- | --- | --- | --- | --- | --- | --- | --- | --- | --- | --- | --- | --- | --- | --- |
| **Antimicrobial Agent** | **Amikacin** | **Carbapenem** | | **Ceftriaxone** | **Ciprofloxacin** | | **Colistin** | **ESBL** | | **Gentamicin** | **MRSA** | | **Vancomycin** |  |
|  | Proportion (95% CI)  (# isolates) | Proportion (95% CI)  (# isolates) | | Proportion (95% CI)  (# isolates) | Proportion (95% CI)  (# isolates) | | Proportion (95% CI)  (# isolates) | Proportion (95% CI)  (# isolates) | | Proportion (95% CI)  (# isolates) | Proportion (95% CI)  (# isolates) | | Proportion (95% CI)  (# isolates) |  |
| ***A. baumannii*** |  |  | |  |  | |  |  | |  |  | |  |  |
|  | - | - | | - | - | | - | - | | - | - | | - |  |
| ***E. coli*** |  |  | |  |  | |  |  | |  |  | |  |  |
|  | - | - | | - | - | | - | - | | - | - | | - |  |
| ***K. pneumoniae*** |  |  | |  |  | |  |  | |  |  | |  |  |
|  | - | - | | - | - | | - | - | | - | - | | - |  |
| ***P. aeruginosa*** |  |  | |  |  | |  |  | |  |  | |  |  |
|  | 0.83  (46) | 0.22  (46) | | - | 0.69 (0.56, 0.80)  (60) | | - | - | | 0.70 (0.57, 0.81)  (60) | - | | - |  |
| ***S. aureus*** |  |  | |  |  | |  |  | |  |  | |  |  |
|  | - | - | | - | 0.40  (30) | | - | - | | 0.13  (30) | 0.56  (34) | | 0.00  (30) |  |
| ***S. epidermidis*** |  |  | |  |  | |  |  | |  |  | |  |  |
|  | - | - | | - | - | | - | - | | - | - | | - |  |
| When combining less than two studies, the biostatistical analysis was limited. Therefore, mean resistance without the 95% CI was reported. | | | | | | | | | | | | | |  |
| *Acinetobacter baumannii* (*A. baumannii*), *Escherichia coli* (*E. coli*), extended-spectrum beta-lactamase (ESBL), *Klebsiella pneumoniae* (*K. pneumoniae*), methicillin-resistant *Staphylococcus aureus* (MRSA), *Pseudomonas aeruginosa* (*P. aeruginosa*), *Staphylococcus aureus* (*S. aureus*), *Staphylococcus epidermidis* (*S. epidermidis*) | | | | | | | | | | | | | |  |
|  | | | | | | | | | | | | | |  |
| **Legend** | | | | | | | | | | | | | |  |
| < 20% Resistance | | | 20-39% Resistance | | | 40-59% Resistance | | | 60-79% Resistance | | | ≥ 80% Resistance | |  |
